# Ozone Reactions with Olefins and Alkynes: Kinetics, Activation Energies, and Mechanisms

**DOI:** 10.1021/acs.est.4c07119

**Published:** 2025-02-28

**Authors:** Yan Wang, Eva M. Rodríguez, Daniel Rentsch, Zhimin Qiang, Urs von Gunten

**Affiliations:** † School of Architecture, Civil and Environmental Engineering (ENAC), École Polytechnique Fédérale de Lausanne (EPFL), Lausanne 1015, Switzerland; ‡ Key Laboratory of Drinking Water Science and Technology, Research Center for Eco-Environmental Sciences, Chinese Academy of Sciences, Beijing 100085, China; § University of Chinese Academy of Sciences, Beijing 100049, China; ∥ Departamento de Ingeniería Química y Química Física, Universidad de Extremadura, Badajoz 06007, Spain; ⊥ Swiss Federal Laboratories for Materials Testing and Research (EMPA), Duebendorf 8600, Switzerland; # School of Environmental Science & Engineering, Shanghai Jiao Tong University, 800 Dongchuan Road Minhang District, Shanghai 200240, China; ¶ Swiss Federal Institute of Aquatic Science and Technology, Eawag, Duebendorf 8600, Switzerland

**Keywords:** ozonation, olefins, alkynes, reaction kinetics, activation energy, transformation mechanism, substituent effect

## Abstract

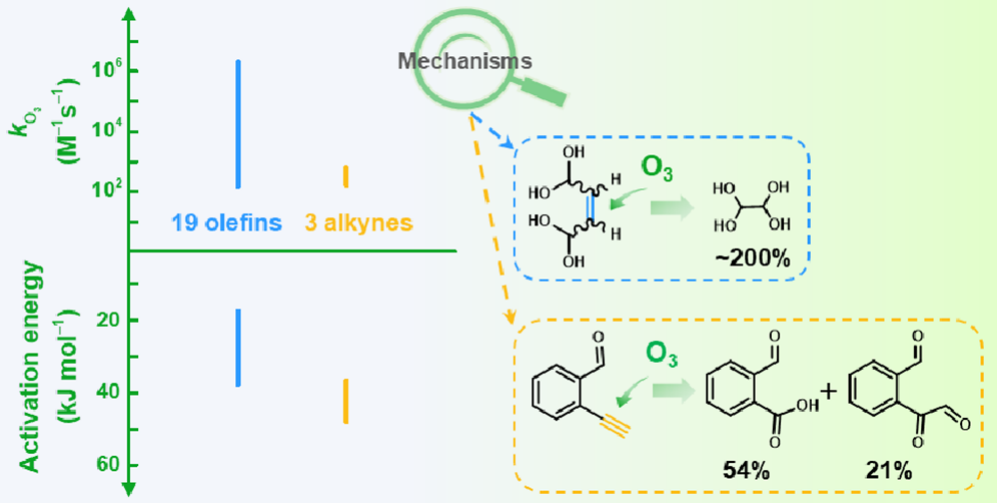

The temperature dependence of the kinetics and the mechanisms of ozone reactions with 19 olefins and 3 alkynes were investigated. The second-order rate constants (*k*_O_3__) for ozone reactions with olefins were mostly in the range of 10^3^–10^6^ M^–1^ s^–1^, with activation energies of 17.4–37.7 kJ mol^–1^. In comparison, alkynes had lower *k*_O_3__ (∼10^2^ M^–1^ s^–1^) and higher activation energies (36.7–48.1 kJ mol^–1^). Reactivities of both olefins and alkynes are mainly influenced by inductive effects of substituents, with steric effects observed for cyclic olefins. 2-Buten-1,4-dial (BDA), synthesized with a novel method, is a toxic olefinic oxidation product from phenols. Its *cis-* and *trans*-isomers show distinct reactivities with ozone, with *k*_O_3__ (20 °C) of 3.0 × 10^3^ and 1.2 × 10^4^ M^–1^ s^–1^, respectively. Two mols of glyoxal were formed per mol of ozonated BDA, with a slow release of the second mol from an α-hydroxyalkylhydroperoxide intermediate. 2-Ethynylbenzaldehyde reacts with ozone with a stoichiometry of 1:1 and *k*_O_3__ (20 °C) = 1.6 × 10^2^ M^–1^ s^–1^. Ozone attacks the ethynyl group, yielding a carboxyl product (2-carboxybenzaldehyde, 54%), an aldehyde product (phthaldialdehyde), and a dicarbonyl product with a stoichiometric release of H_2_O_2_ (21%). This study provides kinetic and mechanistic information for assessing the abatement of olefin- and alkyne-containing micropollutants by ozonation at various temperatures.

## Abstract 2

Effects of substituents and temperature on ozone reaction kinetics with olefins and alkynes were investigated, aiding in assessing efficiencies of abatement of olefin/alkyne-containing micropollutants during ozonation.

## 1 Introduction

Ozone and other chemical oxidants have been widely applied for inactivation of microorganisms and oxidation of pollutants in water treatment.^1−3^ Ozone effectively oxidizes a large variety of organic compounds, and there is a significant body of kinetic and mechanistic information for these reactions.^4^ Ozone reactions with organic compounds typically follow second-order kinetics, first-order in both ozone and the organic compounds.^5^ Despite its effectiveness, ozonation cannot achieve complete mineralization of organic compounds, which results in the formation of transformation products.^4^ Nevertheless, transformation products of bioactive compounds have typically much lower biological activities than their parent compounds.^3,6^

Ozone is an electrophile and preferably attacks electron-rich functional groups (e.g., olefins, activated aromatic compounds, neutral amines, and reduced sulfur compounds).^1^ Natural organic matter contains some olefinic moieties^7^ and algal blooms release olefin-containing compounds (e.g., microcystins^8^ and some taste and odor compounds^9^). Many synthetic organic compounds such as pharmaceuticals,^2^ pesticides,^10,11^ and industrial chemicals^12,13^ also contain olefinic moieties. Reactivities of ozone with olefins vary significantly based on substituents, with second-order rate constants (*k*_O_3__) varying by up to 8 orders of magnitude (10^–1^ to 10^7^ M^–1^ s^–1^).^4^ However, information on the ozone reactivity of olefins with electron-withdrawing substituents remains limited, particularly for α,β-unsaturated carbonyl compounds raising toxicological concerns.^14,15^ Alkynes, another class of unsaturated compounds, also react with ozone.^16,17^ The ethynyl moiety is an important functional group in some micropollutants (e.g., propyzamide and 17α-ethinylestradiol).^16^ The ozonolysis of alkynes in the gas phase and organic solvents has been extensively studied.^17−19^ However, there is limited information about the kinetics and mechanisms of ozone reactions with alkynes in aqueous solutions.

In real treatment systems, temperatures in the range of 1–30 °C can be observed, causing *k*_O_3__ to deviate significantly from standard laboratory conditions at ∼20 °C and thus needing estimations.^20^ So far, information about the temperature dependence (activation energy) of ozone reactions is scarce. Activation energies for ozone consumption in different water types ranged from 65 to 70 kJ mol^–1^,^21^ while for inactivation of microorganisms like *Escherichia coli*, *Bacillus subtilis* (*B. subtilis*) spores, and the bacteriophage MS2 were 37.1,^22^ 42.1,^23^ and 12.2–23.4 kJ mol^–1^ (pH-dependent),^24^ respectively. For ozone reactions with organic compounds with *k*_O_3__ ≤ 10^3^ M^–1^ s^–1^, activation energies mostly ranged from 35 to 50 kJ mol^–1^.^25−27^ In a recent study on abatement of 51 micropollutants in a pilot-scale ozonation of lake water at 6 °C, *k*_O_3__ were calculated based on activation energies for compounds with similar structures, providing a rough estimate.^20^ However, many studies did not consider the temperature for micropollutant abatement during ozonation under realistic conditions. Overall, there is a significant lack of data on the activation energies, especially for ozone reactions with olefins, alkynes, and nitrogen-containing compounds. Activation energies can be calculated using the Arrhenius equation with *k*_O_3__ determined at various temperatures from the slope of a plot of the ln *k*_O_3__ as a function of the temperature (eqs 1 and 2).^28^
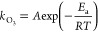
1
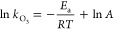
2where *R*, *T*, *E*_a_, and *A* are the universal gas constant (8.3145 J K^–1^ mol^–1^), absolute temperature (K), activation energy (J mol^–1^), and pre-exponential factor (M^–1^ s^–1^), respectively.

The aims of this study were to investigate kinetics, activation energies, and mechanisms for the reactions of ozone with 19 olefins and 3 alkynes. Measurements of *k*_O_3__ were performed in the pH range of 1.7–7.0 for olefins and alkynes depending on their chemical structures. A temperature range of 10–25 °C was chosen for the calculation of activation energies. 2-Buten-1,4-dial (BDA, olefin), a problematic transformation product from oxidation of phenols, and 2-ethynylbenzaldehyde(alkyne) were selected for detailed mechanistic studies.

## 2 Materials and Methods

### 2.1 Chemicals

Chemicals were mostly obtained from commercial suppliers and used without further purification (for details, see Text S1 (Supporting Information)). Ozone stock solutions were prepared and standardized similarly as in previous studies (Text S2).^24^ The hydrolysis of one mol of 2,5-dimethoxy-2,5-dihydrofuran (DMDF) theoretically gives rise to the formation of one mol of BDA with the release of two mols of methanol, where the presence of acid promotes the forward reaction (Scheme S1).^29^ Hence, BDA was synthesized from the hydrolysis of DMDF in the presence of polymer-bound *p*-toluenesulfonic acid (TSA) which can be removed by filtration (Text S3 and Figures S1 and S2). This was necessary to avoid oligomerization with the formation of polymers in the presence of a residual acid.

### 2.2 Characterization of BDA

**Figure 1:**
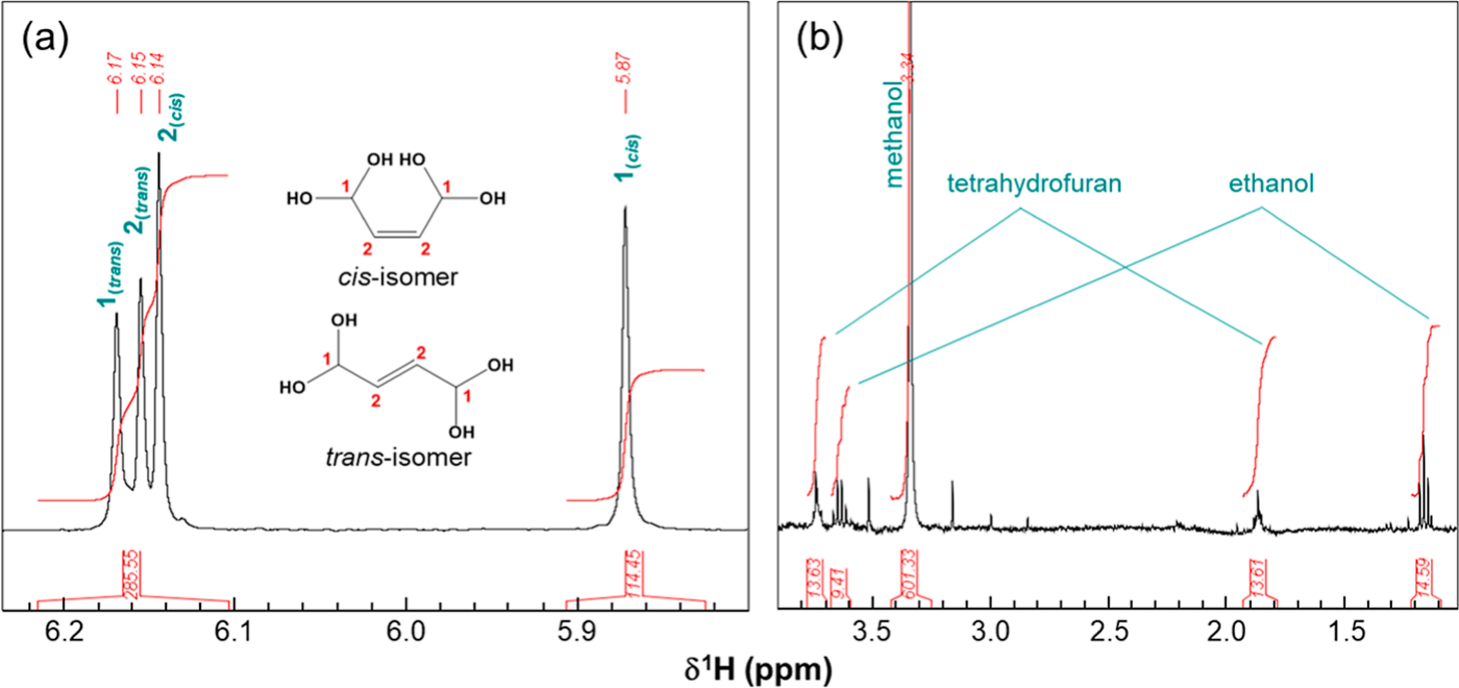
Regions of interest of the ^1^H NMR spectrum for a 1 mM BDA solution. (a) Resonance assignments and chemical structures of *cis*- and *trans*-BDA hydrates (including integrals) and (b) assignments of signals of methanol (mainly resulting from the hydrolysis of 2,5-dimethoxy-2,5-dihydrofuran) and ethanol and tetrahydrofuran (present as residual solvents in the TSA resin).

Figure S3 depicts the cross-signals in the ^1^H–^13^C heteronuclear single-quantum correlation (HSQC) NMR spectrum, which can be assigned to a mixture of the *cis*- and *trans*-isomers of BDA hydrates, in agreement with previous observations.^30^ The relative concentrations of the two isomers can be obtained based on the integrals for the signals of *cis*- and *trans*-isomers (Figure 1a). The signals of methanol (resonance at 3.34 ppm) and traces of ethanol and tetrahydrofuran (THF) were identified (Figure 1b), for which relative concentrations with respect to BDA hydrates can also be calculated (Text S3). As summarized in Table S1, a stable *cis*-isomer:*trans*-isomer ratio of 56%:44% for the BDA hydrates was obtained for five different batches. The synthesis method developed in this study made it possible to obtain a fast and complete conversion to BDA hydrates without the formation of polymeric products. Identical NMR analysis results for different batches of BDA samples indicate that the hydrolysis protocol is reproducible.

### 2.3 Ozone Kinetics

*k*_O_3__ for 19 olefins and 3 alkynes were determined. Among them, *k*_O_3__ for 20 compounds were determined by stopped-flow and for 2 compounds also verified by competition kinetics, while for sorbic alcohol and β-cyclocitral, they were only determined by competition kinetics due to the lack of an appropriate UV absorbance, which is necessary for stopped-flow measurements (Table S2). Tertiary butanol (*t*-BuOH) was used in all experiments to scavenge hydroxyl radicals (^•^OH).^4^ Low pH is favorable to stabilize ozone in aqueous solutions,^4^ and therefore, pH 2.3 ± 0.1 (10 mM phosphate buffer) was commonly applied for kinetics measurement, if not otherwise specified.

Stopped-flow experiments were carried out under pseudo-first-order conditions ([target compound]_0_:[O_3_]_0_ (molar ratio) ≥ 10:1), in the presence of excess *t*-BuOH to scavenge ≥90% ^•^OH by considering the *k*^·^_OH_ for the selected compounds (for details of *t*-BuOH concentrations, see Table S3). *k*_O_3__ values were measured at 10, 15, 20, and 25 (±1) °C to determine activation energies. The apparent second-order rate constants () for olefins and alkynes containing carboxylic acid groups were determined at pH 7.0 and/or other pH (1.7–6.0) to account for the speciation of these compounds. The experimental conditions for each compound are summarized in Table S4. The calculation methods for *k*_O_3__ from the evolution of absorbance at the working wavelength in the stopped-flow spectrophotometer are provided in Text S4.

For competition kinetics,^4,26^ a series of identical reaction solutions containing the target compound and a competitor mostly in the presence of 0.1 M *t*-BuOH were prepared, followed by addition of variable ozone doses (for details, see Figure S4). For 2-ethynylbenzaldehyde, kinetic experiments were performed at pH 7.0 because *k*_O_3__ for the competitor bezafibrate is only available at pH 7.0 in the literature.^26^*k*_O_3__ can be calculated from the relative abatement of the target compound (C) and competitor (P) by plotting ln([C]/[C]_0_) as a function of ln([P]/[P]_0_) according to eq 3.^4^
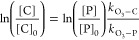
3where [C] and [P], [C]_0_ and [P]_0_, and  and  are instant concentrations, initial concentrations, and *k*_O_3__ for the target compound C and competitor P, respectively.

All experiments were performed at least in duplicate, and the reported data are average values.

### 2.4 Transformation of BDA during Ozonation

To further investigate the reaction of BDA with ozone, the ensuing formation and yield of glyoxal was determined by derivatization with *p*-toluenesulfonyl hydrazide to generate a hydrazone, which can be measured by LC–MS/MS^31,32^ or HPLC (for details, see Text S5). Glyoxal is present in the form of a trimer dihydrate in the commercial standard, and therefore, for the preparation of the glyoxal stock solutions, sufficient equilibration time (≥2 h, Figure S5) is required for the standard reagent to produce monomer-containing solutions (Scheme S2 and Text S6).

### 2.5 Transformation of 2-Ethynylbenzaldehyde during Ozonation

For the identification of transformation products, 200 μM 2-ethynylbenzaldehyde in 10 mM phosphate buffer (pH 2.3) was ozonated with a molar ratio of [O_3_]:[2-ethynylbenzaldehyde] ≤ 1:1 in the presence of 20 mM *t*-BuOH. For the determination of the H_2_O_2_ yield, 500 μM 2-ethynylbenzaldehyde in the presence of 10 mM dimethyl sulfoxide (DMSO) instead of *t*-BuOH as a ^•^OH scavenger was ozonated to avoid H_2_O_2_ formation from the ^•^OH reaction with *t*-BuOH (yield up to 30%^33^ but negligible for DMSO^34^). The formation of H_2_O_2_ was quantified by Allen’s reagent method to avoid interference from organic peroxides (Text S7 and Figure S6).^35^ 2-Carboxybenzaldehyde and phthaldialdehyde are identified as transformation products from the ozonation of 2-ethynylbenzaldehyde, with commercially available standards (for details, see below). Their *k*_O_3__ were further measured to clarify the fate of 2-ethynylbenzaldehyde during ozone reaction, by measurements of the ozone decrease (ozone measurement by the indigo method)^36^ under pseudo-first-order conditions in excess of the target compounds^25,37^ (for details, see Text S8).

### 2.6 Analytical Methods

The synthesized BDA was analyzed by a Bruker AV-III 400 NMR spectrometer (Bruker BioSpin AG, Switzerland).

Kinetics measurements by stopped-flow were performed with an SF-61DX2 stopped-flow instrument (Hitech Scientific), and the data analyses were performed by software installed on the stopped-flow instrument (kinetic studio software).

The identification of transformation products from ozonated 2-ethynylbenzaldehyde was carried out by a Vanquish UHPLC system (Thermo Scientific)-Orbitrap mass analyzer (Orbitrap Exploris 120, Thermo Fisher). More details on the analytical methods are provided in Texts S4 and S9–S11.

## 3 Results and Discussion

### 3.1 Ozone Kinetics

Cinnamic acid (carboxylic acid group, p*K*_a_ = 4.44),^38^ an olefinic compound with well-known *k*_O_3__,^8,39−41^ was used to validate the kinetics measurement system applied in this study. The reactivity of olefins with ozone is pH-independent unless adjacent functional groups undergo acid–base speciation. Species-specific second-order rate constants (20 °C) for cinnamic acid for the neutral and anionic forms were determined as (6.9 ± 0.04) × 10^4^ and (9.0 ± 0.1) × 10^5^ M^–1^ s^–1^, respectively (Table 1). They are consistent with the most recently reported values (20 °C) of 5.8 × 10^4^ and 7.5 × 10^5^ M^–1^ s^–1^, respectively,^8^ as well as a previous *k*_O_3__ at pH 6.5 (22 °C, 7.6 × 10^5^ M^–1^ s^–1^)^24^ (Table S5, with a difference of <20%). Based on this data, the pH dependence of  can be calculated by the fractions of the protonated and deprotonated species and the corresponding species-specific second-order rate constants.^4^

**Table 1:**
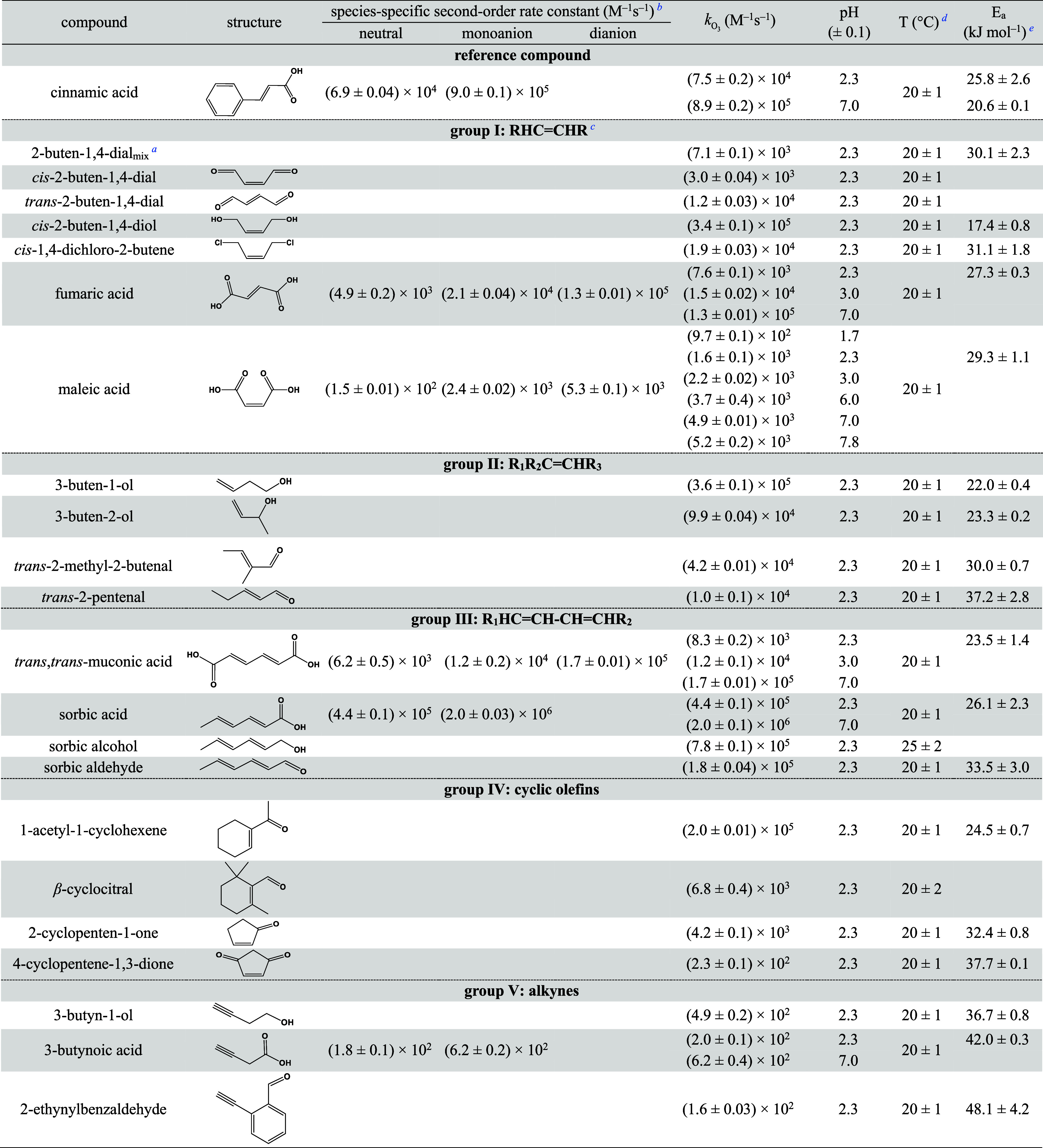
Determined Second-Order Rate Constants (*k*_O_3__) and Activation Energies (E_a_) for the Reactions of Ozone with Selected Olefins and Alkynes (Groups I–V)

a 2-Buten-1,4-dial_mix_: mixture of 56% *cis*- and 44% *trans*-isomers (results for the fractions are shown in Table S1), both present in the form of hydrates in aqueous solution.

b Calculated from the determined *k*_app,O_3__ values at different pHs.

c R: Substituents of olefins.

d T: Temperature for the present *k*_O_3__ determination, which was the same for selected compounds at different pHs.

e *E*_a_ values for cinnamic acid were determined at pH 2.3 (25.8 ± 2.6 kJ mol^–1^) and 7.0 (20.6 ± 0.1 kJ mol^–1^); for all other compounds, they were determined at pH 2.3.

#### 3.1.1 Second-Order Rate Constants for Reactions of Ozone with Target Olefins

Different classes of olefins (as outlined in Table 1) were selected to investigate the effects of carbonyl, carboxyl, and alcohol substituents on their reactivity. The target aliphatic olefins were divided into four groups based on their structural characteristics.

#### Group I: Symmetrically Substituted Mono-olefinic Compounds

A significant dependence of the reactivity on the type of substituent was observed. For example, *k*_O_3__ (20 °C) for *cis*-2-buten-1,4-diol (CH_2_OH substitution) is (3.4 ± 0.1) × 10^5^ M^–1^ s^–1^, which is 2 orders of magnitude higher than for *cis*-2-buten-1,4-dial (*cis*-BDA, CHO substitution, (3.0 ± 0.04) × 10^3^ M^–1^ s^–1^, Table 1). Since ozone preferentially attacks electron-rich moieties, an electron-withdrawing substituent typically leads to a decreasing reactivity of olefinic compounds. Taft σ* constants are commonly applied descriptors for inductive effects of substituents.^42^ A –CH_2_OH group as in *cis*-2-buten-1,4-diol exhibits a lower electron-withdrawing effect than –CHO as in *cis*-BDA, with the corresponding Taft σ* values of 0.62 and 2.15, respectively.^42^ A higher ozone reactivity of *cis*-2-butene-1,4-diol compared to that of *cis*-BDA is in line with this concept. For *cis*-1,4-dichloro-2-butene, the –CH_2_Cl group has an electron-withdrawing effect (σ* = 1.05) between –CH_2_OH and –CHO, which is reflected in an intermediate *k*_O_3__ ((1.9 ± 0.03) × 10^4^ M^–1^ s^–1^, Table 1). Different  values for maleic acid^43−46^ and fumaric acid^43,46,47^ were reported in the literature (Table S5). The obtained  values (20 °C) for maleic acid of (1.3 ± 0.01) × 10^3^ (calculated, pH 2.0), (3.7 ± 0.4) × 10^3^ (pH 6.0, Table 1), and (4.9 ± 0.01) × 10^3^ M^–1^ s^–1^ (pH 7.0, Table 1) and for fumaric acid of (6.3 ± 0.1) × 10^3^ (calculated, pH 2.0), (1.1 ± 0.01) × 10^5^ (calculated, pH 5.0), and (1.3 ± 0.01) × 10^5^ M^–1^ s^–1^ (pH 7.0, Table 1) differ by less than a factor of 2 from most previously reported values^43,47^ and calculated values from the reported species-specific second-order rate constants (Table S5).^46^ Their species-specific second-order rate constants follow the order of dianion > monoanion > acid, consistent with the expected induction effect of the carboxylic acid group.^48,49^Figure S7 shows the measured and calculated pH dependence of the  values of maleic acid. The good agreement between the experiments and calculations confirms the validity of this approach. Maleic acid and fumaric acid are stereoisomers (*cis* and *trans*, respectively), but fumaric acid is 1 order of magnitude more reactive than maleic acid. A similar phenomenon was also observed for 1,2-dichloroethene^25,27^ and 1,1,3,4-tetrachloro-1,3-butadiene^50^ (Table S5). This could be partially explained by the *cis* effect, an observation that the *cis*-isomer is more stable than the corresponding *trans*-isomer.^51,52^ In the case of monoanions, the lower reactivity of maleic acid compared to fumaric acid could be due to the generation of intramolecular hydrogen bonds promoting its stability.^53^

#### Group II. Asymmetrically Substituted Mono-olefinic Compounds

The determined *k*_O_3__ values at 20 °C for group II compounds are in the range of 10^4^–10^5^ M^–1^ s^–1^. *k*_O_3__ for *trans*-2-methyl-2-butenal ((4.2 ± 0.01) × 10^4^ M^–1^ s^–1^, Table 1) differed by a factor of 2 compared to the reported value for methacrolein (1.9 × 10^4^ M^–1^ s^–1^,^54^Table S5). This was attributed to an additional –CH_3_ group at the olefin in *trans*-2-methyl-2-butenal, which leads to a higher electron density. *k*_O_3__ for methacrolein^54^ (Table S5) was nearly a factor of 2 higher than for *trans*-2-pentenal ((1.0 ± 0.1) × 10^4^ M^–1^ s^–1^, Table 1). This is likely due to the α position of the –CH_3_ group for methacrolein compared to the –CH_2_CH_3_ group in the β position for *trans*-2-pentenal. Other evidence for the effect of positions of substituents on the compound’s reactivities is provided by comparing 3-buten-1-ol and 3-buten-2-ol. The distance of the electron-withdrawing −OH group from the double bond decreases in the order of 3-buten-1-ol >3-buten-2-ol, which is reflected in a consistent trend of *k*_O_3__ being (3.6 ± 0.1) × 10^5^ M^–1^ s^–1^ (Table 1) and (9.9 ± 0.04) × 10^4^ M^–1^ s^–1^ (Table 1; previously reported values 7.9 × 10^4^^27^ and 9.1 × 10^4^ M^–1^ s^–1^,^55^Table S5), respectively.

##### Group III: Olefins with Conjugated Double Bonds

*trans*,*trans*-Muconic acid was used as a representative compound of this group, and the stoichiometry for the ozone reaction was demonstrated to be 1 for molar ratios of ozone:*trans*,*trans*-muconic acid <1 (Figure S8). Since there are two olefinic groups, the second double bond also reacts in excess of ozone. Muconic acid exists in three stereoisomers: *trans*,*trans*-muconic acid, *cis*,*trans*-muconic acid, and *cis*,*cis*-muconic acid. Unfortunately, the species-specific second-order rate constants for the *cis*,*trans*- and *cis*,*cis*-isomers are unavailable, and therefore, a comparison of ozone reactivities for the respective species of the three isomers is impossible. For *trans*,*trans*-muconic acid, which has carboxylic acid groups at both ends, its species-specific second-order rate constants are about 1–2 orders of magnitude lower than those of sorbic acid, which has a –CH_3_ group at one end instead (Table 1). This difference is consistent with the electron-withdrawing effect of the carboxylic acid group compared with the electron-donating effect of the –CH_3_ group. Sorbic alcohol, sorbic acid, and sorbic aldehyde have the same structural features with H_3_C–CH=CH–CH=CH-R, where R is –CH_2_OH, –COOH, and –CHO, with *k*_O_3__ at pH 2.3 (25 °C) being (7.8 ± 0.1) × 10^5^ (Table 1), (5.2 ± 0.04) × 10^5^ (Table S4), and (2.2 ± 0.1) × 10^5^ M^–1^ s^–1^ (Table S4), respectively. These reactivities are in line with the electron-withdrawing effects of the substituents R which increase in the order of –CH_2_OH (Taft σ* = 0.62) < –COOH (2.08) < –CHO (2.15). The  for sorbic acid at pH 3.0 and 8.0 were previously reported to be 3.2 × 10^5^ and 9.6 × 10^5^ M^–1^ s^–1^ (Table S5),^56^ respectively, using an earlier published  for cinnamic acid as a competitor.^39^ After correction with  values for cinnamic acid determined in this study (Table 1), the  for sorbic acid at pH 3.0 and 8.0 could be updated to 4.8 × 10^5^ and 2.3 × 10^6^ M^–1^ s^–1^, respectively. This is very close to the  values at pH 3 and 8 obtained in the current study being (4.7 ± 0.1) × 10^5^ and (2.0 ± 0.03) × 10^6^ M^–1^ s^–1^, respectively (calculated from the determined species-specific second-order rate constants for sorbic acid, Table 1).

##### Group IV: Cyclic Olefins

There is only limited information on the reactivities of this class of compounds.^9,57,58^ As for the open-chain olefins, substituents in the α position have a pronounced effect on the ozone reactivities. Cyclohexene (2.2 × 10^6^ M^–1^ s^–1^ at 25 °C,^57^Table S5) is about 1 order of magnitude more reactive than 1-acetyl-1-cyclohexene ((2.3 ± 0.04) × 10^5^ M^–1^ s^–1^ at 25 °C, Table S4), attributed to the electron-withdrawing effect of the –CO–CH_3_ group on the latter. 2-Cyclopenten-1-one and 4-cyclopentene-1,3-dione with one and two carbonyl groups in the α position, respectively, differ in their reactivities with ozone by about 1 order of magnitude, with *k*_O_3__ = (4.2 ± 0.1) × 10^3^ and (2.3 ± 0.1) × 10^2^ M^–1^ s^–1^, respectively (20 °C, Table 1). As shown in Table S6, cyclohexene, 1-acetyl-1-cyclohexene, and β-cyclocitral contain the basic olefinic structures of ethene, methyl vinyl ketone, and 2-butenal, respectively. Relative to ethene (1.8 × 10^5^ M^–1^ s^–1^)^27^ and methyl vinyl ketone (3.7 × 10^4^ M^–1^ s^–1^),^54^ the *k*_O_3__ values of cyclohexene (2.2 × 10^6^ M^–1^ s^–1^)^57^ and 1-acetyl-1-cyclohexene ((2.0 ± 0.01) × 10^5^ M^–1^ s^–1^, this study) are higher by factors of 12 and 5, respectively. This is likely due to the electron-donating effect of the adjacent alkyl groups in the case of the ring structures. However, relative to 2-butenal (*k*_O_3__ not measured but approximated by *trans*-2-methyl-2-butenal, (4.2 ± 0.01) × 10^4^ M^–1^ s^–1^), the *k*_O_3__ for β-cyclocitral ((6.8 ± 0.4) × 10^3^ M^–1^ s^–1^, Table 1; previously reported value 3.9 × 10^3^ M^–1^ s^–1^,^9^Table S5) is lower by a factor of 6–11. The large difference in *k*_O_3__ is possibly caused by the steric hindrance of an ozone attack on the ring structure of β-cyclocitral. A 4 orders of magnitude decrease in *k*_O_3__ caused by the steric effect has been observed for endrin (<2.0 × 10^–2^ M^–1^ s^–1^,^37^Table S5) compared to *cis*-1,2-dichloroethene ((3.1–8.0) × 10^2^ M^–1^ s^–1^,^25,27,37^Table S5).

The electronic effects discussed above for noncyclic olefins (groups I–III) also apply to heterocyclic olefins (Table S7). Addition of electron-donating groups (−CH_3_) to the carbon–carbon double bond as in 4-methylimidazole (protonated, 1.7 × 10^3^ M^–1^ s^–1^)^45^ increases the *k*_O_3__ compared to imidazole (protonated, 2.2 × 10^1^ M^–1^ s^–1^^43^–1.5 × 10^3^ M^–1^ s^–1^^59^). Conversely, the addition of electron-withdrawing –CO groups in maleimide (4.2 × 10^3^ M^–1^ s^–1^)^59^ significantly decreases the *k*_O_3__ when compared to pyrrole (8.6 × 10^5^ M^–1^ s^–1^).^59^ A similar pattern was observed for furans. At pH 7, the degree of electron withdrawal by the carboxylic acid group attached to the furan ring, with –COO^–^ as the predominant form because of a p*K*_a_ < 4.4,^60,61^ follows this sequence: 3-(2-furyl) propanoic acid < 2-furoic acid < furan-2,5-dicarboxylic acid, and consequently, the  values show the opposite tendency (3.2 × 10^6^, 5.9 × 10^5^, and 8.5 × 10^4^ M^–1^ s^–1^, respectively, Table S7).^62^

Taft σ* constants have been previously applied to develop quantitative structure–activity relationships (QSAR) for predicting *k*_O_3__ of organic compounds containing ozone-reactive functional groups such as olefins.^63^ However, Taft σ* constants are not always available; for instance, the values for groups like –CH=CH–CH_2_OH in sorbic alcohol and for cyclic structures remain undefined. Moreover, Taft σ* constants have limitations, such as their inability to distinguish stereoisomers (e.g., maleic acid and fumaric acid, which share identical Taft σ* constants despite their distinct stereochemistry). These constraints hindered the development of a comprehensive QSAR model to all the measured olefins in this study.

#### 3.1.2 Second-Order Rate Constants for Reactions of Ozone with Target Alkynes

*k*_O_3__ values for the three selected alkynes at 20 °C were determined to be in the range of (1.6 ± 0.03) × 10^2^–(6.2 ± 0.4) × 10^2^ M^–1^ s^–1^ (Table 1). This is similar to reported *k*_O_3__ for 1-ethinyl-1-cyclohexanol (2 × 10^2^ M^–1^ s^–1^,^16^Table S5). *k*_O_3__ values for the reactions of ozone with alkynes (∼10^2^ M^–1^ s^–1^) are significantly lower than those for olefins (mostly 10^3^–10^6^ M^–1^ s^–1^, see above). For 3-butyn-1-ol as a representative alkyne, *k*_O_3__ is (4.9 ± 0.2) × 10^2^ M^–1^ s^–1^ (Table 1), which is 3 orders of magnitude lower than for the corresponding olefin 3-buten-1-ol ((3.6 ± 0.1) × 10^5^ M^–1^ s^–1^, Table 1). It may be surprising to observe this pattern because the electron density in alkynes is also high. One possible explanation could be the stronger π bonds in alkynes than olefins, as reflected by the shorter carbon–carbon triple bond length compared to carbon–carbon double bond length,^64^ which has been verified by calculations using acetylene and ethene as examples.^65^ Because of an acid–base speciation of 3-butynoic acid (p*K*_a_ = 3.62,^66^ predicted), the species-specific second-order rate constants (20 °C) were determined to be (1.8 ± 0.1) × 10^2^ and (6.2 ± 0.2) × 10^2^ M^–1^ s^–1^ for the protonated and deprotonated forms, respectively (Table 1). The effect of substituents on the alkyne reactivity was tested and *k*_O_3__ increased in the order of 3-butynoic acid (acid, (1.8 ± 0.1) × 10^2^ M^–1^ s^–1^) < 3-butyn-1-ol ((4.9 ± 0.2) × 10^2^ M^–1^ s^–1^) (Table 1). This is consistent with the electron-withdrawing effect of the substituents: –COOH (Taft σ* = 2.08) > –CH_2_OH (0.62).^42^ In the case of 1-ethinyl-1-cyclohexanol, the –OH group was assumed to show a higher inductive effect than that of the six-membered ring. The –OH group of 1-ethinyl-1-cyclohexanol (*k*_O_3__ = 2 × 10^2^ M^–1^ s^–1^,^16^Table S5) was closer to the alkyne group than for 3-butyn-1-ol (*k*_O_3__ = (4.9 ± 0.2) × 10^2^ M^–1^ s^–1^, Table 1), resulting in a higher electron-withdrawing effect with an ensuing lower reactivity with ozone.

### 3.2 Activation Energies for Reactions of Ozone with Olefins and Alkynes

Changes in temperature affect not only second-order rate constants but also the speciation of compounds with acidic functional groups. For cinnamic acid as an example, according to the linear form of the van’t Hoff equation (eq 4),^67^ its p*K*_a_ at 10 °C was calculated to be 4.63 after plotting the ln(*K*_a_) against 1/*T* in the temperature range of 15–25 °C reported previously (respective p*K*_a_ = 4.59, 4.53, and 4.50 at 15, 20, and 25 °C).^68^
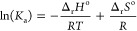
4where Δ_r_*H*^o^ and Δ_r_*S*^o^ are the standard reaction enthalpy (J mol^–1^) and standard reaction entropy (J K^–1^ mol^–1^), respectively.

The species distribution of cinnamic acid at pH 2.3 was calculated to be constant with a proportion of the neutral form ranging from 0.995 to 0.993, as the temperature changed from 10 to 25 °C. In this case,  of cinnamic acid at pH 2.3 gradually increased from (5.2 ± 0.1) × 10^4^ M^–1^ s^–1^ at 10 °C to (9.0 ± 0.8) × 10^4^ M^–1^ s^–1^ at 25 °C (Table S4) and the effect of p*K*_a_ change can be neglected.

Based on this, a correlation between ln *k*_O_3__ and the inverse of absolute temperature can be made to obtain the activation energy (eq 2). The activation energy was calculated as 25.8 ± 2.6 kJ mol^–1^ for the ozone reaction with cinnamic acid (Figure S9). Since pH affects the  for olefins with substituents with acid–base speciation, the activation energy for cinnamic acid was also determined at pH 7.0 to be 20.6 ± 0.1 kJ mol^–1^ (Table 1), which is in agreement with the reported values of 21.2 kJ mol^–1^ at pH 6.5^24^ and 19.1 kJ mol^–1^ at pH 7.2^8^ (Table S5). This indicates that changes in the acid–base speciation do affect not only the  but also the activation energies.

Table 1 reports the activation energies for ozone reactions with selected olefins and alkynes. The minimum and maximum activation energies for the reactions of ozone with olefins were determined to be 17.4 ± 0.8 kJ mol^–1^ for *cis*-2-buten-1,4-diol and 37.7 ± 0.1 kJ mol^–1^ for 4-cyclopentene-1,3-dione, respectively. These results are consistent with previously reported activation energies for ozone reactions with olefins (18.0–35.1 kJ mol^–1^, Table S5). Overall, *k*_O_3__ values for olefins at 25 °C are about a factor of 1.4–2.3 higher than at 10 °C. The activation energies for the ozone reactions with alkynes are significantly higher than for olefins and ranged from 36.7 to 48.1 kJ mol^–1^. *k*_O_3__ values for alkynes at 25 °C are about a factor of 2.2–2.8 higher than at 10 °C.

The activation energies for olefinic compounds determined in this and previous studies were plotted as a function of the logarithm of *k*_O_3__ (Figure S10). There is a moderate correlation between *k*_O_3__ and the activation energy. For most of the selected olefins in this study, activation energies are within a 95% confidence interval of literature-reported *k*_O_3__. Overall, an order of magnitude increase in *k*_O_3__ was observed for a decrease in the activation energy of about 5 kJ mol^–1^.

**Figure 2:**
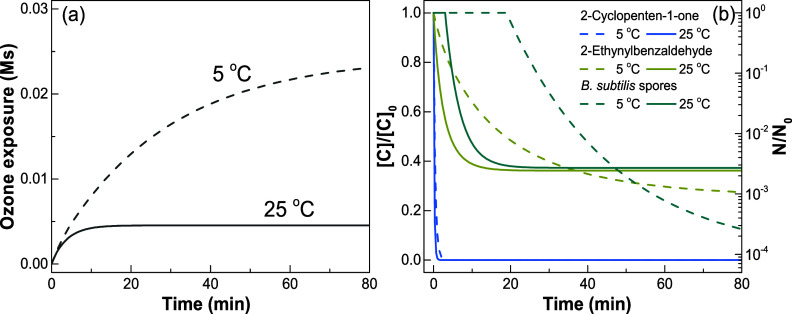
Calculated effects of temperature (5 and 25 °C) on (a) ozone exposure and (b) abatement of 2-cyclopenten-1-one and 2-ethynylbenzaldehyde and the inactivation of *B. subtilis* spores in Lake Zurich water. [C]/[C]_0_ represents the relative residual concentration of the target compound during ozonation, and N/N_0_ represents the relative residual numbers of *B. subtilis* spores during ozonation. Calculation conditions: [O_3_]_0_ = 1 mg L^–1^. Detailed kinetic information for the ozone decay, the abatement of 2-cyclopenten-1-one and 2-ethynylbenzaldehyde, and the inactivation of *B. subtilis* spores, as well as the calculation methods are provided in Text S12.

To gain more insights into the variations in ozonation efficacy pertaining to the inactivation of microorganisms and oxidation of organic compounds at different temperatures, the effects of temperature (5 and 25 °C) on ozone exposure, the abatement of 2-cyclopenten-1-one and 2-ethynylbenzaldehyde, and the inactivation of *B. subtilis* spores^23^ were modeled for ozonation of Lake Zurich water^21^ (Text S12 and Figure 2). Figure 2a shows the cumulative ozone exposure as a function of reaction time. Ozone is much less stable at 25 °C, with first-order rate constants for its decrease in the initial and secondary phases of 5.2 × 10^–3^ s^–1^ and 4.4 × 10^–3^ s^–1^, respectively, compared to 5 °C (1.9 × 10^–3^ s^–1^ and 6.0 × 10^–4^ s^–1^, respectively),^21^ with an ensuing lower ozone exposure at 25 °C (maximum: 4.5 × 10^–3^ Ms) than at 5 °C (maximum: 2.3 × 10^–2^ Ms). Nevertheless, the abatement of 2-cyclopenten-1-one at both temperatures is quite similar. This is due to the offsetting effect of reduced ozone exposure at higher temperature by the increase of *k*_O_3__ of 2-cyclopenten-1-one under these conditions. For 2-ethynylbenzaldehyde with a much lower *k*_O_3__ ((2.2 ± 0.01) × 10^2^ M^–1^ s^–1^ at 25 °C, this study, Table S4), the relative abatement is better at lower temperature for residence times >37 min. The calculated inactivation of *B. subtilis* spores is more efficient at higher temperatures, achieving 2-log inactivation for a contact time of 9 min at 25 °C and 38 min at 5 °C, respectively. This is mainly due to the increased lag phase at lower temperatures, which for higher ozone exposures is no longer critical anymore. However, due to the higher ozone exposure at 5 °C, the inactivation of *B. subtilis* spores continuously proceeded, yielding 3-log inactivation for a contact time of 57 min. Overall, this shows that the water temperature has a significant influence on the efficiency of ozonation processes. For target compounds with intermediate–high *k*_O_3__, the efficiency is similar or better at the higher temperature (e.g., olefins), whereas for slowly reacting targets, the trend may be reversed (e.g., alkynes). For the inactivation of microorganisms, both the inactivation rate constants and a potential lag phase are decisive. The simulation does not provide exact transformation/inactivation data but rather trends, which illustrate the importance of considering temperature changes when going from the laboratory to realistic systems.

### 3.3 Transformation of BDA during Ozonation

Oxidative treatments (e.g., chlorination, UV photolysis, and radical oxidation) of phenolic compounds can lead to the formation of BDA,^69,70^ which is problematic due to the potential genotoxicity and mutagenicity of α,β-unsaturated carbonyls.^15^ Even though this compound may be (partially) abated in biological processes,^31^ its reactivity with ozone is still of great interest. The lack of a commercial standard makes it difficult to obtain mechanistic information about BDA transformation during ozonation. Hence, BDA was synthesized and characterized (see details in Sections 2.1 and 2.2) before its abatement kinetics and mechanisms were investigated.

#### 3.3.1 Kinetics of BDA Oxidation by Ozone

As discussed above, both *cis*- and *trans*-isomers of BDA hydrates are present in the final BDA samples (Figure 1). To gain insights about the reactivities of the two isomers, two batches of kinetic experiments were conducted, as described in Text S13. For the first batch, a *k*_O_3__ of (7.1 ± 0.1) × 10^3^ M^–1^ s^–1^ was obtained from the original BDA solution, containing 56% *cis*- and 44% *trans*-BDA. For the second batch, a preozonated BDA sample was prepared with a molar BDA:ozone ratio of 2:1. A *k*_O_3__ of (4.1 ± 0.1) × 10^3^ M^–1^ s^–1^ was obtained from the preozonated BDA solution (containing a calculated fraction of 88% *cis*- and 12% *trans*-BDA). On the basis of the different fractions of the *cis*- and *trans*-isomers during two batches of *k*_O_3__ measurements, individual *k*_O_3__ values can be calculated. Considering the *cis* effect (see above),^51,52^*k*_O_3__ for the *cis*- and *trans*-isomers were calculated to be (3.0 ± 0.04) × 10^3^ and (1.2 ± 0.03) × 10^4^ M^–1^ s^–1^, respectively (Text S13).

#### 3.3.2 Glyoxal Formation during Ozonation of BDA

**Figure 3:**
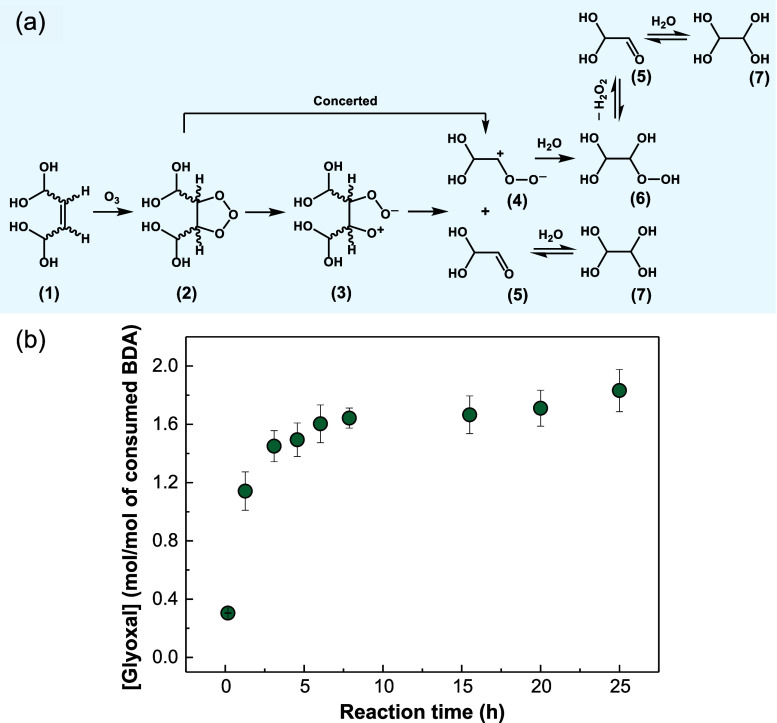
Glyoxal formation during ozonation of BDA. (a) General Criegee-type mechanism for the reaction of ozone with the *cis*- and *trans*-isomers of BDA. The wavy bonds stand for the two possible stereoisomers. (b) Glyoxal formation as a function of time after complete depletion of ozone (<1 min). Experimental conditions: [BDA]_0_ = 30 μM, [O_3_]_0_ = 6 and 12 μM for two experiments, [*t*-BuOH]_0_ = 3 mM, pH 2.3, 10 mM phosphate buffer. The BDA was synthesized following the procedure described in Text S3. The circles represent the average values, and the error bars represent the range of the measured values for the two ozone doses.

Ozone typically reacts with olefins by the Criegee mechanism.^1^ The corresponding transformation pathway for the *cis*- and *trans*-BDA hydrates is shown in Figure 3a. The ozonide (**2**) formed from the cycloaddition of ozone to BDA decomposes to form one mol of glyoxal (**7**) and one mol of a zwitterion (**4**) and the hydrated zwitterion (**6**) decomposes to another mol of glyoxal (**6** ⇌ **5** ⇌ **7**). Overall, two mols of glyoxal are expected to be formed per mol of ozonated BDA. The glyoxal yield as a function of the time increases gradually within 25 h (Figure 3b). At 25 h contact time, 1.84 mol of glyoxal per mol of dosed ozone was determined, which is close to the theoretical factor of 2. Due to uncertainties in ozone dosing, an estimated error of 10% in terms of the glyoxal yield is possible. Under the applied experimental conditions, ozone is completely depleted within 1 min because *k*_O_3__ for both *cis*- and *trans*-isomers of BDA hydrates are >10^3^ M^–1^ s^–1^ (see above). The reversible reactions between the glyoxal trimer and hydrated monomer during preparation of the glyoxal stock solution can also be a potential source of error (Text S6). A similar pattern of glyoxal formation as a function of holding time was also observed when the BDA solution was prepared differently (Figure S11). One mol of glyoxal per mol of consumed BDA was formed relatively fast within about 1 h, which can be interpreted by the decomposition of the Criegee ozonide (**2** → **5**, Figure 3a). The formation of the second mol of glyoxal takes about 24 h, which can be explained by the decomposition of a relatively stable α-hydroxyalkylhydroperoxide.^71^

### 3.4 Transformation of 2-Ethynylbenzaldehyde during Ozonation

#### 3.4.1 Kinetics of the Reaction of Ozone with 2-Ethynylbenzaldehyde

**Figure 4:**
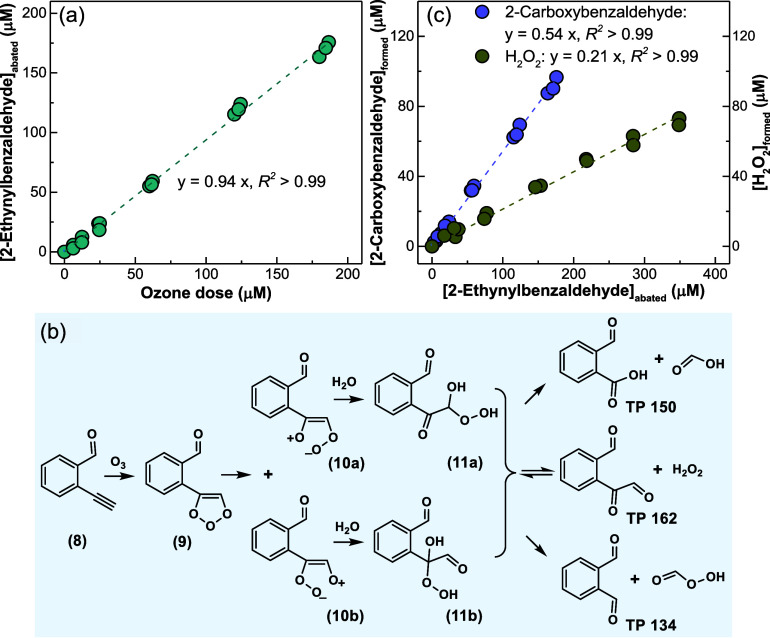
Reaction of ozone with 2-ethynylbenzaldehyde. (a) Stoichiometry of the reaction, (b) proposed transformation mechanism, and (c) 2-carboxybenzaldehyde (TP 150) and H_2_O_2_ formation as functions of the abated 2-ethynylbenzaldehyde. Experimental conditions for stoichiometry and 2-carboxybenzaldehyde yield: [2-ethynylbenzaldehyde]_0_ = 200 μM, [O_3_]_0_ = 0–186 μM, [*t*-BuOH]_0_ = 20 mM, pH 2.3, 10 mM phosphate buffer. Experimental conditions for H_2_O_2_ yield: [2-ethynylbenzaldehyde]_0_ = 500 μM, [O_3_]_0_ = 0–640 μM, [DMSO]_0_ = 10 mM, pH 2.3, 10 mM phosphate buffer.

Figure 4a shows a linear correlation of the abatement of 2-ethynylbenzaldehyde as a function of the ozone dose with a slope = 0.94, indicating a reaction stoichiometry close to 1. As discussed above, the *k*_O_3__ values for aliphatic alkynes are on the order of 10^2^ M^–1^ s^–1^. For 2-ethynylbenzaldehyde, *k*_O_3__ (25 °C, stopped-flow) of (2.2 ± 0.01) × 10^2^ M^–1^ s^–1^ (Table S4) and an activation energy of 48.1 ± 4.2 kJ mol^–1^ (Table 1) have been determined. The reactivity of the compound was further examined through competition kinetics using bezafibrate as a competitor, resulting in a *k*_O_3__ of (2.0 ± 0.1) × 10^2^ M^–1^ s^–1^ (25 ± 2 °C, Figure S4d), consistent with the value determined via the stopped-flow method. The reactivity of the benzaldehyde component can be disregarded, as it reportedly reacts only slowly with ozone (2.5 M^–1^ s^–1^).^25^ Based on these observations, it can be assumed that for 2-ethynylbenzaldehyde, the ozone attack occurs primarily at the ethynyl group, as expected.

#### 3.4.2 Determination of Transformation Products from the Reaction of Ozone with 2-Ethynylbenzaldehyde

Figure 4b shows the proposed transformation pathway for the ozone attack at the ethynyl group of 2-ethynylbenzaldehyde. Ozonation of alkynes is generally assumed to occur similarly to a Criegee-type mechanism.^17^ The first step is a cycloaddition to generate an ozonide (**9**) which further decomposes to the zwitterions (**10a** and/or **10b**). Subsequently, the alkoxyalkyl hydroperoxides (**11a** and/or **11b**) are potentially formed via hydrolysis reactions. Three transformation products with *m*/*z* of 150 (TP 150), 162 (TP 162), and 134 (TP 134), respectively, were identified using an Orbitrap LC–MS/MS (Text S14). The chemical structure of TP 150 (Figure S12a) and TP 134 (Figure S12b) was confirmed via comparison with commercial standards of 2-carboxybenzaldehyde (Figure S12e) and phthaldialdehyde (Figure S12f), respectively. TP 162, despite the lack of a standard, was proposed as a dicarbonyl compound based on the MS^2^ spectrum obtained in the positive ionization mode (Figure S12c), the formation of which occurred with a release of H_2_O_2_. Upon ozonation of 1-ethinyl-1-cyclohexanol, the formation of a corresponding dicarbonyl compound and carboxylic acid compound was also observed previously.^16^

As shown in Figure 4c, the yield of 2-carboxybenzaldehyde (TP 150) was determined to be 54% with respect to the abated 2-ethynylbenzaldehyde. Quantification of phthaldialdehyde (TP 134) was not possible due to the low sensitivity of the HPLC analytical method for this compound. For H_2_O_2_ quantification, 20 times molar excess of DMSO was used (no H_2_O_2_ formation in contrast to *t*-BuOH).^33^ Due to its much higher *k*_O_3__ (most recently reported value of 4.8 M^–1^ s^–1^)^72^ compared to *t*-BuOH (0.003 M^–1^ s^–1^),^25^ DMSO competed for ozone with 2-ethynylbenzaldehyde, with the ozone consumption calculated to be 38% and ensuing 62% by 2-ethynylbenzaldehyde. Consistently, 0.57 mol of 2-ethynylbenzaldehyde was abated per mole of ozone (Figure S13a). Meanwhile, 0.54 mol of 2-carboxybenzaldehyde was detected per mol of consumed 2-ethynylbenzaldehyde (Figure S13b) which is the same as in Figure 4c and indicates that the change of the scavenger did not affect the transformation mechanism. An average H_2_O_2_ yield of 21% based on the transformed 2-ethynylbenzaldehyde over the entire ozonation time from 1 to 75 h (Figure S13c) was determined (Figure 4c). Since one mol of H_2_O_2_ is released per mol of TP 162 formed, its yield is assumed to be 21% based on the abated 2-ethynylbenzaldehyde. Therefore, the tentatively identified transformation products accounted for about 75% of the abated 2-ethynylbenzaldehyde (54% 2-carboxybenzaldehyde and 21% dicarbonyl compound TP 162).

#### 3.4.3 Reactivities of Representative Transformation Products with Ozone

As shown in Figure S14, *k*_O_3__ for 2-carboxybenzaldehyde and phthaldialdehyde at pH 2.3 were determined to be 0.16 and 1.15 M^–1^ s^–1^, respectively. Considering a p*K*_a_ of 4.57 for 2-carboxybenzaldehyde,^73^ the measured second-order rate constant represents the species-specific second-order rate constant for the neutral form. Both transformation products are significantly less reactive than the parent compound, and therefore, they do not interfere with determination of the second-order rate constant for the ethynyl moiety in the parent compound and are also not significantly further degraded via ozone reactions. Nevertheless, further products can be formed due to ^•^OH reactions during ozonation of real waters.^20,74^

## 4 Practical Implications

The measured *k*_O_3__ values for olefins generally aligned with the reported data and followed the expected substituent effects. Water temperature is often unreported in the literature, allowing only rough comparisons between the measured and reported *k*_O_3__. While olefins are electron-rich and typically susceptible to ozone attack, the presence of carbonyl, carboxyl, and alcohol groups significantly reduced their reactivities with ozone. Nevertheless, the measured *k*_O_3__ for such olefins are generally higher than those of extensively studied chlorinated olefins due to a stronger electron-withdrawing effect of chlorine. *k*_O_3__ for alkynes were determined to be approximately 10^2^ M^–1^ s^–1^.

Seasonal changes in the water temperature can affect the efficiency of ozonation processes significantly. For olefins with high *k*_O_3__ and low activation energies, changes in water temperature and hence ozone exposures have a limited effect on their abatement efficiency. In contrast, for alkynes with low *k*_O_3__ and high activation energies, a lower water temperature likely enhances their abatement if the residence times allow full ozone depletion. For the inactivation of *B. subtilis* spores at lower water temperature, the increased lag phase and reduced inactivation rate constant both lead to lower inactivation efficiency at the early stage of ozonation, while higher ozone exposure enhances inactivation at a later stage.

To achieve a 2-log inactivation of *B. subtilis* spores, ozone exposures of 4.1 × 10^–3^ Ms at 25 °C and 1.8 × 10^–2^ Ms at 5 °C are required, equivalent to ozone doses of about 0.91 and 0.75 mg L^–1^ in Lake Zurich water (dissolved organic carbon concentration: 1.3 mg L^–1^),^21^ respectively. Under these conditions, highly reactive compounds (e.g., olefins with *k*_O_3__ ≥ 2 × 10^3^ M^–1^ s^–1^) can be completely abated (>99.9%) by ozone at both temperatures, while alkynes with *k*_O_3__ ≈ 10^2^ M^–1^ s^–1^ are only partially abated. For instance, 2-ethynylbenzaldehyde was calculated to be abated by 59% and 64% at 25 and 5 °C, respectively. Compounds with low ozone reactivities (i.e., *k*_O_3__ ≤ 1 M^–1^ s^–1^) are barely affected (<2% abatement). Even though ^•^OH have limited impact on inactivation of *B. subtilis* spores, they could enhance the abatement of compounds with moderate and low ozone reactivities. This can be exemplified by increases in relative abatements of 59% to 68% for 2-ethynylbenzaldehyde and of 0.4% to 22% for a compound with *k*_O_3__ = 1 M^–1^ s^–1^ if ^•^OH reactions are also included, assuming a ^•^OH:ozone exposure ratio (*R*_ct_) of 10^–8^ and *k*^•^_OH_ of 6 × 10^9^ M^–1^ s^–1^ at 25 °C.

Various oxidative treatments of phenols lead to the formation of BDA. Despite the relatively high reactivity of the BDA isomers with ozone (*k*_O_3__ > 10^3^ M^–1^ s^–1^), this compound was still detected in a previous study during ozonation of wastewater for a relatively high specific ozone dose (≤3 mgO_3_/mgC).^31^ This suggests that the BDA formation is not completed at this point. The analysis of oxidation products reveals that the ozone reactions with olefins and alkynes primarily follow a Criegee-type mechanism, with minor alternative pathways reported in few cases.^46^ The carbonyl product, glyoxal, formed from BDA ozonation, was observed to be effectively removed by biological post-treatment processes,^75^ as was BDA,^31^ and therefore, biological barriers as implemented after ozonation allow mitigation of these undesired compounds. For alkynes, carbonyl and carboxyl moieties are also formed on the core structure of the ethynyl group, and it is expected that these compounds are at least partially biodegradable.^20^

## Supporting Information Available

Reagents and solutions, details on reaction kinetics and analytical methods, measurement methods for target compounds and transformation products, synthesis and NMR analysis of BDA, and summary of second-order rate constants (PDF)
