# Supplementary material for: Ozone Reactions with Olefins and Alkynes: Kinetics, Activation Energies, and Mechanisms
Source: Environ Sci Technol. 2025 Feb 28;59(9):4733–44. doi: 10.1021/acs.est.4c07119 (PMC11912337; doi:10.1021/acs.est.4c07119)
Supplement: Supplementary file 1 — es4c07119_si_001.pdf [file es4c07119_si_001.pdf]

Supporting Information

Ozone Reactions with Olefins and Alkynes: Kinetics, Activation Energies, and Mechanisms

Yan Wang,[†,‡,§] Eva M. Rodríguez,[†,∥] Daniel Rentsch,[⊥]
Zhimin Qiang,[#] Urs von Gunten[*,†,¶]

[†] School of Architecture, Civil and Environmental Engineering (ENAC), École Polytechnique Fédérale de Lausanne (EPFL), Lausanne 1015, Switzerland

[‡] Key Laboratory of Drinking Water Science and Technology, Research Center for Eco-Environmental Sciences, Chinese Academy of Sciences, Beijing 100085, China

[§] University of Chinese Academy of Sciences, Beijing 100049, China

[∥] Departamento de Ingeniería Química y Química Física, Universidad de Extremadura, Universidad de Extremadura, Badajoz 06007, Spain

[⊥] Swiss Federal Laboratories for Materials Testing and Research (EMPA), Duebendorf 8600, Switzerland

[#] School of Environmental Science & Engineering, Shanghai Jiao Tong University, 800 Dongchuan Road, Minhang District, Shanghai 200240, China

[¶] Eawag, Swiss Federal Institute of Aquatic Science and Technology, Duebendorf 8600, Switzerland

* Corresponding authors:
(Urs von Gunten) E-mail: urs.vongunten@eawag.ch. Phone: + 41-58-765-5270

60 pages including 14 Texts, 7 Tables, 14 Figures, and 2 Schemes.

CONTENTS

Text S1. Chemicals S5

Text S2. Preparation and standardization of ozone stock solution S6

Text S3. Details of BDA synthesis method...S6

Text S4. Details of stopped-flow spectrophotometric method for determination of second-order rate constants for the reactions of the target compounds with ozone S7

Text S5. Experimental details for measurement of the glyoxal yield from ozonated BDA S9

Text S6. Preparation of a glyoxal solution..S10

Text S7. Allen's reagent method for H_2O_2 quantification...S10

Text S8. Experimental details for determination of second-order rate constants for the reactions of 2-carboxybenzaldehyde and phthaldialdehyde with ozone...................S11

Text S9. Analytical method to identify BDA by NMR spectroscopy S12

Text S10. HPLC methods for measurement of competition kinetics, and quantification of glyoxal hydrazone, 2-carboxybenzaldehyde, and 2-ethynylbenzaldehyde S12

Text S11. Analytical method to identify the transformation products formed from ozonation of 2-ethynylbenzaldehyde by UHPLC-Orbitrap MS S14

Text S12. Calculation of the ozonation efficiency for abatement of 2-cyclopenten-1-one and 2-ethynylbenzaldehyde and inactivation of *B. subtilis* spores at 5 and 25 °C in Lake Zurich water S15

Text S13. Measurement of the second-order rate constants for reactions of ozone with *cis*- and *trans*-isomers of BDA S16

Text S14. Identification of the transformation products (TP 150, TP 162, and TP 134) formed from ozonation of 2-ethynylbenzaldehyde..S18

Table S1. Fractions of *cis*- and *trans*-isomers of BDA hydrates for different rinsing cycles..S19

Table S2. Methods for the determination of second-order rate constants for the reactions of ozone with olefins and alkynes (groups I-V) at pH 2.3 or other indicated pH values S20

Table S3. Second-order rate constants ($k_{^{\bullet}OH}$) for the reactions of $^{\bullet}OH$ with selected olefins and alkynes and the corresponding reference compounds............................S22

Table S4. Temperature dependence of second-order rate constants (k_{O_3}) for the reactions of ozone with selected olefins and alkynes (groups I-V) determined by

stopped-flow in this study (experimental conditions are also shown).......................S23

Table S5. Summary of previously reported second-order rate constants (k_{O_3}) and activation energies (E_a) for the reactions of ozone with olefins and alkynes S32

Table S6. Second-order rate constants (k_{O_3}) for the reactions of ozone with cyclic olefins and the corresponding non-cyclic analogues S38

Table S7. Examples for reported second-order rate constants (k_{O_3}) for the reactions of ozone with heterocycles with an olefinic moiety as the dominant reactive site S39

Figure S1. Regions of interest of the ^{1}H NMR spectra of (a) 1 M DMDF hydrolyzed for two weeks, (b) 1.2 M DMDF hydrolyzed for several minutes in the presence of 0.16 M DCl, (c) 1.2 M DMDF in the presence of 0.20 M NaOD, and (d) 1.2 M DMDF (H_2O:D_2O = 9:1). S40

Figure S2. ^{1}H NMR spectra of a 1.2 M DMDF solution hydrolyzed in the presence of 0.16 M DCl recorded after (a) several minutes, and (b) 12 h (additional application of a weak air stream to remove methanol for approximately 1 h), and (c) 3 days. The resonances of the BDA main product of the three spectra are normalized to the same intensities. S41

Figure S3. Characterization of BDA species by NMR spectroscopy: Region of interest of ^{1}H-^{13}C HSQC NMR spectrum with chemical structures and resonance assignments of a 1 mM BDA solution (H_2O:D_2O = 9:1). S41

Figure S4. Competition kinetics plots for the determination of second-order rate constants (k_{O_3}) for the reactions of ozone with (a) *trans*-2-pentenal using cinnamic acid as a competitor (n = 2), (b) sorbic alcohol using sorbic acid as a competitor (n = 3), (c) *β*-cyclocitral using *trans*-2-pentenal as a competitor (n = 2), and (d) 2-ethynylbenzalhyde (2-EBA) using bezafibrare as a competitor (n = 2). S42

Figure S5. Effect of equilibration time for an aqueous glyoxal stock solution on the signal intensity (HPLC-UV (305 nm)) of glyoxal hydrazone. S43

Figure S6. Determination of H_2O_2 concentrations formed during ozonation of 2-ethynylbenzaldehyde by Allen's reagent. (a) UV absorbance change at 351 nm (A_{351}) for determination of a H_2O_2 standard and an ozonated 2-ethynylbenzaldehyde sample by the Allen's reagent method, and (b) a plot of A_{351} as a function of the H_2O_2 concentration in the presence of 2-ethynylbenzaldehyde. S44

Figure S7. Speciation of maleic acid (mole fraction, solid lines) and the apparent second-order rate constants (k_{app, O_3} , circles (experiments) and dashed line (model calculation)) for its reaction with ozone as a function of the pH. S45

Figure S8. Evolution of UV spectra for ozonated *trans*,*trans*-muconic acid (MA) samples for different molar ozone:target compound ratios. S46

Figure S9. Arrhenius plots for the determination of activation energies for the reactions of ozone with selected olefins and alkynes. (a) Cinnamic acid at pH 2.3 and 7.0, (b) olefins of group I, (c) olefins of group II, (d) olefins of group III, (e) olefins of group IV, and (f) alkynes of group V (Table 1, main text). S47

Figure S10. Previously reported (stars) and currently determined activation energies (E_a) for the reactions of ozone with olefins (circles) and alkynes (triangles) as a function of the logarithm of the corresponding second-order rate constants (k_{O_3}). S48

Figure S11. Glyoxal formation from ozonation of BDA as a function of time after complete ozone depletion. S49

Figure S12. MS^2 spectra of the identified compounds with an Orbitrap LC-MS/MS. (a) TP 150, (b) TP 134, and (c) TP 162 in an ozonated 2-ethynylbenzaldehyde sample. (d) 2-Ethynylbenzaldehyde, (e) 2-carboxybenzaldehyde, and (f) phthaldialdehyde in their standard mixtures.. ....S52

Figure S13. Ozonation of 2-ethynylbenzaldehyde. (a) Abatement of 2-ethynylbenzaldehyde as a function of the ozone dose, (b) formation of 2-carboxybenzaldehyde as a function of the abated 2-ethynylbenzaldehyde, and (c) H_2O_2 formed as a function of the abated 2-ethynylbenzaldehyde.....................................S53

Figure S14. Reactions of ozone with 2-carboxybenzaldehyde (2-CBA) and phthaldialdehyde (PTA). Logarithms of relative ozone residual concentrations in excess of (a) 2-CBA and (b) PTA as functions of time at pH 2.3. Plots of k_{obs} of ozone consumption *versus* the initial concentrations of (c) 2-CBA and (d) PTA. S54

Scheme S1. BDA hydrates formation from DMDF hydrolysis. S55

Scheme S2. (1) Hydration and (2, 3) di/trimerization reaction of glyoxal in aqueous solutions. S55

REFERENCES S56

Text S1. Chemicals

Cinnamic acid (≥ 99%), 2,5-dimethoxy-2,5-dihydrofuran (DMDF, mixture of *cis* and *trans*, 97%), *cis*-2-buten-1,4-diol (97%), *cis*-1,4-dichloro-2-butene (95%), fumaric acid (≥ 99.0%), maleic acid (≥ 99%), 3-buten-1-ol (≥ 98.0%), 3-buten-2-ol (97%), *trans*-2-methyl-2-butenal (≥ 99%), *trans*-2-pentenal (95%), *trans*,*trans*-muconic acid (98%), sorbic acid (≥ 99.0%), sorbic alcohol (≥ 97%), sorbic aldehyde (95%), 1-acetyl-1-cyclohexene (97%), *β*-cyclocitral (analytical standard), 2-cyclopenten-1-one (98%), 4-cyclopentene-1,3-dione (95%), 3-butyn-1-ol (97%), 3-butynoic acid (95%), 2-ethynylbenzaldehyde (97%), bezafibrate (≥ 98.0%), glyoxal (40% w/w in water), 2-carboxybenzaldehyde (97%), phthaldialdehyde (≥ 99%), sodium phosphate dibasic (≥ 99.0%), tertiary butanol (*t*-BuOH, ≥ 99.7%), potassium indigotrisulfonate, dimethyl sulfoxide (DMSO, ≥ 99.9%), *p*-toluenesulfonic acid (TSA, polymer bound, 30–60 mesh, 2.0–3.0 mmol g^{-1} loading), *p*-toluenesulfonyl hydrazide (97%), hydrogen peroxide (30%, stabilized), potassium iodide (≥ 99.5%), and sodium hydroxide (≥ 98%) were all purchased from Sigma Aldrich. Hydrochloric acid (32%) and ammonium molybdate tetrahydrate (81.0–83.0% MoO_3 basis) were obtained from Merck. Potassium hydrogen phthalate (99.5%), acetonitrile (ultra-gradient grade), and *ortho*-phosphoric acid (85%) were obtained from Fluka, Carlo Erba Reagents, and Supelco, respectively.

4-Cyclopentene-1,3-dione was pre-ozonated with ozone with a molar fraction of 5% with respect to 4-cyclopentene-1,3-dione to remove the impurities before the measurement of k_{O_3}. When a 4-cyclopentene-1,3-dione solution was ozonated with an ozone molar ratio of ≤ 10%, a two-phase reaction kinetics was observed from measurement of the ozone decrease by stopped-flow, with the fitting k_{O_3} (25 °C) of 1.6 × 10^4 and 3.9 × 10^2 $M^{-1}s^{-1}$ for the first and second phases, respectively. Using the pre-ozonated 4-cyclopentene-1,3-dione solution (prepared as aforementioned) as a working solution and ozonating it again with an ozone molar ratio of ≤ 10%, a one-phase reaction kinetics was observed, with the fitting k_{O_3} (25 °C) of (3.0 ± 0.02) × 10^2 $M^{-1}s^{-1}$, close to the second-phase observed without pre-ozonation.

Apart from *p*-toluenesulfonyl hydrazide (10 mM) dissolved in acetonitrile, and *β*-cyclocitral and bezafibrate dissolved in a mixture of *t*-BuOH and ultra-purified water, all working solutions were prepared in ultra-purified water from a Milli-Q purification system (Millipore, 18.2 MΩ cm).

Text S2. Preparation and standardization of ozone stock solution

Ozone was produced by purging an ozone/oxygen gas mixture from a CMG 3-5 ozone generator (Innovatech, Rheinbach, Germany) into 1 L of ice-cooled water for at least 1.5 h. The ozone concentration of the stock solution ranged from 1.0–1.4 mM. The ozone concentration of the stock solution was measured spectrophotometrically by a UV-1800 spectrophotometer (Shimadzu) at 260 nm ($\varepsilon_{260\ nm}$ = 3200 $M^{-1}cm^{-1}$).[1]

Text S3. Details of BDA synthesis method

Figure S1a shows the region of interest of the ^{1}H NMR spectrum of a solution of DMDF that was aged in water for two weeks. After the addition of deuterated hydrochloric acid (DCl) to a freshly prepared DMDF solution, a hydrolysis reaction led to the same ^{1}H NMR spectrum within minutes (Figure S1b). In contrast, under basic conditions (Figure S1c), no change in the ^{1}H NMR resonances with respect to the spectrum of the DMDF solution (Figure S1d) was observed over several hours. Figure S2a shows the same spectrum as in Figure S1b. It is notable that with aging of the solution for 12 hours (Figure S2b) or 3 days (Figure S2c), in addition to the resonances of the main BDA products (3.2–3.6 and 5.8–6.3 ppm), broader signals emerge, indicative of oligomers and polymers of BDA. In conclusion, the presence of acid was favorable for the BDA formation, but also promoted oligomerization reactions. Initial experiments using TSA beads (polymer-bound, 2.0–3.0 mmol g^{-1}) showed encouraging results, as the acid could be easily removed through filtration.

Hence, the setup for the preparation of a 1 mM hydrated BDA solution was developed as follows: A stock solution of 20 mM DMDF was prepared in ultra-purified

water and allowed for equilibration for 5 min. A 1 mM DMDF solution was obtained by diluting the stock solution, and to 14 mL of this solution 1.0 g TSA was added. Before use, TSA was rinsed by ultra-purified water 6 times (volume of ultra-purified water:mass of TSA = 10 mL:1.0 g). The DMDF-TSA mixture was kept at room temperature under magnetic stirring for 3 h to allow complete hydrolysis. After this contact time, the TSA was removed by a 0.45 μm syringe filter (CHROMAFIL®Xtra H-PTFE-45/25, Macherey-Nagel, Germany).

Using the signals in Figure 1 as examples for a 1 mM BDA solution, the relative release of ethanol, and THF from TSA after 6 times rinsing with ultra-purified water were 4.8, and 3.4 mol-%, respectively compared to the total concentration of BDA hydrates. When DMDF hydrolysis was performed with TSA without rinsing, much higher concentrations of methanol, ethanol, and THF were observed. The initial concentration of DMDF, the TSA dose, and contact time played important roles in the conversion of DMDF to BDA hydrates and the oligomerization of BDA hydrates (data not shown).

Text S4. Details of stopped-flow spectrophotometric method for determination of second-order rate constants for the reactions of the target compounds with ozone

The applied stopped-flow system (SF-61DX2, Hitech Scientific) included a deuterium lamp power supply unit, a stepped support unit, a control unit, a sample handling unit, and a temperature control unit. The TgK Scientific Kinetic Studio 4.0 was used to control the program of the stopped-flow system and analyze the kinetic data.

For second-order processes, ozonation kinetics can be expressed as eq S1.

$$\frac{d[O_3]}{dt} = \frac{d[C]}{dt} = -k_{O_3}[O_3][C] \qquad (S1)$$

where C represents the tested olefin (or alkyne); $[O_3]$ and [C] (M) are the instant concentrations of ozone and tested olefin (or alkyne), respectively; t (s) means the reaction time; and k_{O_3} ($M^{-1}s^{-1}$) is the second-order rate constant.

All experiments for k_{O_3} determination by stopped-flow were performed in molar excess of olefins (or alkynes) relative to ozone ([olefin]$_0$ (or [alkyne]$_0$):[O_3]$_0$ $\geq$ 10:1). In this case, the abatement of olefins (or alkynes) was maximally 10% resulting in pseudo-first-order conditions. Eq S1 can be then simplified to eq S2.

$$\frac{d[O_3]}{dt} = \frac{d[C]}{dt} = -k_{obs}[O_3] \quad \text{(S2)}$$

where k_{obs} (s^{-1}) is the observed pseudo-first-order rate constant.

As summarized in Table S2, mostly the decrease of ozone was monitored in excess of the target compound under pseudo-first-order conditions on the stopped-flow spectrophotometer to calculate the k_{O_3} .The natural logarithm of the relative residual ozone concentration can be plotted as a function of t (s) to obtain k_{obs} (eq S3):

$$ln\frac{[O_3]_t}{[O_3]_0} = -k_{obs}t \quad \text{(S3)}$$

where $[O_3]_t$ and $[O_3]_0$ (M) are the residual ozone concentrations at reaction time of t and at t_0, respectively.

In a few cases, the abatement of olefins/alkynes was monitored to calculate the k_{O_3} , because the working wavelength on the stopped-flow spectrophotometer to monitor the ozone decrease was not feasible, due to a significant overlap of the olefins/alkynes spectra. When the decrease of the tested olefin (or alkyne) concentration was followed to obtain k_{obs}, the instant ozone concentration can be calculated as follows if a 1:1 stoichiometry is assumed (eq S4):

$$[O_3] = [O_3]_0 - ([C]_0 - [C]) = [O_3]_0 - [C]_0 + [C] \quad \text{(S4)}$$

where $[C]_0$ (M) is the initial concentration of the tested olefin (or alkyne). Thereby $[O_3]_0$ – $[C]_0$ is a constant.

Then the kinetics can be expressed by eqs S5 and S6.

$$\frac{d[C]}{dt} = -k_{obs}[O_3] = -k_{obs}([O_3]_0 - [C]_0 + [C]) \quad \text{(S5)}$$

$$ln\frac{([O_3]_0 - [C]_0) + [C]_t}{[O_3]_0} = -k_{obs}t \quad \text{(S6)}$$

where $[C]_t$ is the residual concentration the tested olefin (or alkyne) at reaction time of

t.

Based on the evolution of absorbance (y) at a certain wavelength as a function of the reaction time (t), k_{obs} can be fitted by the Kinetic Studio 4.0 software using $y = -\mathrm{A}\,\mathrm{e}^{-k_{obs}t} + \mathrm{B}$. According to eqs S3 and S6, A and B are constant values only related to the initial concentrations of ozone and/or the tested compound but independent on the reaction time. The values of k_{obs}, A, and B can be directly obtained as output from the software. It should be noted that the formation of oxidation products from the ozone reactions with olefins/alkynes could cause interference in the monitored UV absorbance on the stopped-flow spectrophotometer. Therefore, the UV spectra for a series of ozonated samples prepared from addition of variable ozone doses to identical solutions containing the target olefins/alkynes were measured in advance, to select a working wavelength for which the spectral interference caused by the oxidation products can be minimized.

Text S5. Experimental details for measurement of the glyoxal yield from ozonated BDA

The analytical procedure for the measurement of the glyoxal yield from ozonated BDA included 3 steps: (1) ozonation of BDA, (2) derivatization of ozonated BDA sample; and (3) HPLC analysis of glyoxal hydrazone.

(1) Ozonation of BDA. Two ozonation experiments were conducted: 30 μM BDA in the presence of 3 mM *t*-BuOH and 10 mM phosphate buffer (pH 2.3) in 20 mL amber vials were dosed with 6 and 12 μM ozone, respectively. The total volume of sample after ozone dosing was set to 20 mL to prevent ozone loss to the headspace. Based on the high reactivity of BDA with ozone (discussed in detail below), ozone is completely consumed in tenths of seconds, and consequently, no ozone quencher was added to the ozonated samples.

(2) Derivatization of ozonated BDA sample. At each preset reaction time (10 min–25 h), 1.36 mL of ozonated sample was withdrawn and immediately derivatized by

mixing with 30 μL of 10 mM *p*-toluenesulfonyl hydrazide and 30 μL of 1.0 M HCl.

(3) HPLC analysis of glyoxal hydrazone. After derivatization for 10 min, the samples were injected into an HPLC system (Ultimate 3000, Thermo Scientific) to determine glyoxal hydrazone.

Text S6. Preparation of a glyoxal solution

Glyoxal is generally present in the form of hydrates in aqueous solution, and it is prone to undergo dimerization and even higher order oligomerization reactions depending on the concentration (Scheme S2). The commercial glyoxal standard was present in the form of trimers composed of 3 mols of glyoxal and 2 mols of water. When the glyoxal concentration is lower than 1 M, the hydrated monomer is the predominant species.[2,3] As shown in Figure S5, it took about 2 h for a glyoxal stock solution (0.84 mM) to get fully equilibrated. This means that the concentration and equilibration time should be taken into account when preparing stock solutions of such types of carbonyl compounds. The addition of acid does not strongly affect the oligomerization reaction of glyoxal.[4] On the basis of these considerations, the monomer of the dihydrate was assumed to be the glyoxal species present in the ozonated BDA sample.

Text S7. Allen's reagent method for H_2O_2 quantification

The Allen's reagent method has been used to separately quantify H_2O_2 and organic peroxides formed from ozonation reactions based on reaction kinetics.[5] H_2O_2 and organic peroxides react with molybdate-catalyzed I^- to generate I_3^- in a similar manner. The yield of I_3^- can be spectrophotometrically measured at 351 nm with a $\varepsilon_{351\ nm}$ of 25000 $M^{-1}cm^{-1}$.[5,6] The second-order rate constant for H_2O_2 (2.5 $M^{-1}s^{-1}$) is much higher than that of most organic peroxides. Hence, the Allen's reagent method can distinguish H_2O_2 from organic peroxides formed during ozonation of 2-ethynylbenzaldehyde.

Two aqueous solutions were prepared in advance for use, including solution 1 containing 295 mM potassium hydrogen phthalate, and solution 2 containing a mixture

of 100 mM potassium iodide, 50 mM sodium hydroxide, and 40 μM ammonium molybdate tetrahydrate.[5] The procedure was as follows: 1 mL of sample was first mixed with 0.5 mL of solution 2 and the absorbance at 351 nm (A_{351}) was recorded. Then 0.5 mL of solution 1 was added and immediately mixed. The evolution of A_{351} of the final mixture was recorded over time.

A calibration curve of H_2O_2 standards in a non-ozonated 2-ethynylbenzaldehyde matrix was firstly conducted using the Allen's reagent method. The H_2O_2 stock solution was freshly standardized by a UV-1800 spectrophotometer (Shimadzu) at 240 nm ($\varepsilon_{240\ nm}$ = 40 $M^{-1}cm^{-1}$).[7] As shown in Figure S6a, A_{351} reached a plateau when recorded for 100 s. Therefore, the A_{351} at 100 s *versus* the H_2O_2 concentration was plotted for the calibration curve. As shown in Figure S6b, a good linear calibration was observed for H_2O_2 concentrations in the range of 5–100 μM and it fitted well with the expected results. In terms of the ozonated 2-ethynylbenzaldehyde sample, the evolution of A_{351} as a function of time was consistent with that of the H_2O_2 standard (Figure S6a). Therefore, A_{351} at 100 s is applicable to measure the H_2O_2 yield from ozonation of 2-ethynylbenzaldehyde.

Text S8. Experimental details for determination of second-order rate constants for the reactions of 2-carboxybenzaldehyde and phthaldialdehyde with ozone

The experiments were carried out in a 250 mL screw-top flask with magnetic stirring, fitted with a dispenser system.[8] The target compound (3.0–9.0 mM for 2-carboxybenzaldehyde and 1.0–3.5 mM for phthaldialdehyde) in the presence of 10 times molar excess of *t*-BuOH in phosphate buffer (pH 2.3, 10 mM) was dosed with ozone ($[O_3]$:[target compound](molar ratio) ≤ 1:10). At preset reaction times (20 s – 20 min for 2-carboxybenzaldehyde and 20 s – 15 min for phthaldialdehyde), an aliquot of the sample was taken by the dispenser system and added to an amber vial containing the indigo reagent, which was prepared via dissolving 1 mM potassium indigotrisulfonate in 20 mM phosphoric acid.[9] After rapid mixing, the absorbance at 600 nm was measured.[8,9] The ozone consumption by the target compound was corrected

after subtracting the ozone decay in the absence of the target compound.

Text S9. Analytical method to identify BDA by NMR spectroscopy

The ^{1}H and ^{1}H-^{13}C HSQC NMR spectra were recorded on a Bruker AV-III 400 spectrometer (Bruker BioSpin AG, Switzerland) using a 5 mm CryoProbe™ Prodigy probe at frequencies of 400.2 (^{1}H) and 100.6 (^{13}C) MHz. All NMR experiments were performed with 540 µL of the BDA aqueous solutions thoroughly mixed with 60 µL of D_2O added for lock and shimming at 298 K. The ^{1}H NMR spectra were recorded applying the Bruker noesypr1d water suppression pulse sequence and the ^{1}H-^{13}C HSQC NMR data was recorded with water suppression as well during the recycle time. ^{1}H and ^{13}C NMR chemical shifts (δ) were calibrated to the signal of methanol at 3.34 and 49.5 ppm, respectively.[10]

Text S10. HPLC methods for measurement of competition kinetics, and quantification of glyoxal hydrazone, 2-carboxybenzaldehyde, and 2-ethynylbenzaldehyde

HPLC with a UV detector was operated with a Nucleosil 100-5 C18 column (l × i.d. = 125 mm × 3.0 mm, particle size = 5 µm). A binary mobile phase comprised of (A) ultra-purified water containing 10 mM H_3PO_4 (pH 2.3) and (B) pure acetonitrile, was always applied. Detailed operation parameters vary for the different compounds.

Competition kinetics for ozonation of *trans*-2-pentenal using cinnamic acid as a competitor: Both, cinnamic acid and *trans*-2-pentenal were eluted with an isocratic mobile phase (70% A and 30% B) with a flow rate of 1.0 mL min^{-1}, and detected at 254 nm at 2.9 min and 245 nm at 1.7 min, respectively. The limits of quantification (LOQs) for cinnamic acid and *trans*-2-pentenal were 2.7 µM and 22 µM, with standard deviations of 0.12 µM and 0.29 µM, respectively. The method showed good linearity in the tested range of 5–150 µM for cinnamic acid and 50–350 µM for *trans*-2-pentenal.

Competition kinetics for ozonation of sorbic alcohol using sorbic acid as a

competitor: Both, sorbic acid and sorbic alcohol were eluted with an isocratic mobile phase (70% A and 30% B) with a flow rate of 1.0 mL min^{-1} and detected at 220 nm and 263 nm. Sorbic acid and sorbic alcohol appeared at the same retention time (1.7 min). Compared to sorbic acid, the peak intensity of sorbic alcohol at 263 nm can be neglected (< 5%). Hence, the sorbic acid concentration can be first obtained from the peak area at 263 nm, then its contribution to the peak area at 220 nm can be calculated. As a result, the sorbic alcohol concentration can be calculated from the peak area at 220 nm after subtracting the contribution of sorbic acid. The LOQs for sorbic acid and sorbic alcohol were 2.5 μM and 1.4 μM, with standard deviations of 0.26 μM and 0.14 μM, respectively. The method showed good linearity in the tested range of 2.5–100 μM for both sorbic acid and sorbic alcohol.

Competition kinetics for ozonation of β-cyclocitral using *trans*-2-pentenal as a competitor: Both, *trans*-2-pentenal and β-cyclocitral were eluted with an isocratic mobile phase (60% A and 40% B) with a flow rate of 1.0 mL min^{-1} and detected at 220 nm at 1.5 min and 254 nm at 9.1 min, respectively. The LOQs for *trans*-2-pentenal and β-cyclocitral were 9.6 μM and 4.3 μM, with standard deviations of 0.71 μM and 0.21 μM, respectively. The method showed good linearity in the tested range of 12.5–150 μM for *trans*-2-pentenal and 5–300 μM for β-cyclocitral.

Competition kinetics for ozonation of 2-ethynylbenzaldehyde using bezafibrate as a competitor: Both, bezafibrate and 2-ethynylbenzaldehyde were eluted with a gradient mobile phase (0.0 min, 70% A and 30% B; 10.0 min, 30% A and 70% B; and 12.1 min, 70% A and 30% B) with a flow rate of 0.8 mL min^{-1} and detected at 220 nm at 9.3 min and 5.2 min, respectively. The LOQs for bezafibrate and 2-ethynylbenzaldehyde were 0.35 μM and 0.18 μM, with standard deviations of 0.033 μM and 0.025 μM, respectively. The method showed good linearity in the tested range of 1–50 μM for both bezafibrate and 2-ethynylbenzalhyde.

Quantification of glyoxal hydrazone: Glyoxal hydrazone was eluted with a gradient mobile phase (0.0 min, 60% A and 40% B; 6.0 min, 40% A and 60% B; 10.0 min, 5% A and 95% B; and 16.0 min, 60% A and 40% B) with a flow rate of 0.5 mL min^{-1} and detected at 305 nm at 7.9 min. The LOQ for glyoxal hydrazone was 1.9 μM

with a standard deviation of 0.0067 μM. The method showed good linearity in the tested range of 2.5–30 μM.

Quantification of 2-carboxybenzaldehyde formation and 2-ethynylbenzaldehyde abatement during ozonation of 2-ethynylbenzaldehyde: Both, 2-carboxybenzaldehyde and 2-ethynylbenzaldehyde were eluted with a gradient mobile phase (0.0 min, 80% A and 20% B; 13.0 min, 70% A and 30% B; 13.5 min, 60% A and 40% B; and 17.0 min, 80% A and 20% B) with a flow rate of 0.5 mL min^{-1} and detected at 220 nm at 3.5 min and 17.6 min, respectively. The LOQs for 2-carboxybenzaldehyde and 2-ethynylbenzaldehyde were 3.3 μM and 7.9 μM, with standard deviations of 0.17 μM and 1.70 μM, respectively. The method showed good linearity in the tested range of 5–200 μM for 2-carboxybenzaldehyde and 10–200 μM for 2-ethynylbenzalhyde.

Text S11. Analytical method to identify the transformation products formed from ozonation of 2-ethynylbenzaldehyde by UHPLC-Orbitrap MS

The UHPLC system (Vanquish, Thermo Scientific)-Orbitrap mass analyzer (Orbitrap Exploris 120, Thermo Fisher) was equipped with a Waters C18 column (l × i.d. = 100 mm × 2.1 mm, particle size = 1.8 μm). For the UHPLC system, the mobile phase was a mixture of A (95% ultra-purified water + 5% methanol + 0.1% ammonium formate) and B (5% ultra-purified water + 95% methanol + 0.1% ammonium formate), and the gradient elution program (min, % B) was set as (0.5, 5), (8, 95), (10, 95), and (14, 95), with a flow rate of 0.4 mL min^{-1}. For the orbitrap mass analyzer, the ion source type was atmospheric-pressure chemical ionization (APCI); the polarity was both in positive and negative modes; the orbitrap resolution was 120,000 and the full scan range was set as 50–250 m/z.

Text S12. Calculation of the ozonation efficiency for abatement of 2-cyclopenten-1-one and 2-ethynylbenzaldehyde and inactivation of *B. subtilis* spores at 5 and 25 °C in Lake Zurich water

The ozone dose was set as 1 mg L^{-1} and its decrease kinetics in Lake Zurich water reported from a previous study was used to calculate the ozone exposure.[11] In this study,[11] the ozone decrease kinetics generally exhibited two first-order phases, an initial, fast phase followed by a secondary, principal phase. The first-order rate constants for the initial ($k_{dec.1}$) and secondary phases ($k_{dec.2}$) of ozone decrease were 1.9 × 10^{-3} s^{-1} and 6.0 × 10^{-4} s^{-1} at 5 °C, and 5.2 × 10^{-3} s^{-1} and 4.4 × 10^{-3} s^{-1} at 25 °C, respectively.[11] The time points at which the two phases change (t_b) were estimated to be 5 min at 5 °C and 1 min at 25 °C, respectively. Based on the ozone decrease, the ozone exposure (CT), the integrated area under the ozone concentration ($[O_3]$, M) curve when it is plotted as a function of reaction time (t, s), was calculated for 5 and 25 °C using eqs S7 and S8.

$$\int_0^t [O_3]dt = \frac{[O_3]_0}{k_{dec.1}}(1-\exp(-k_{dec.1}t)), \text{ if } t \le t_b \quad \text{(S7)}$$

$$\begin{aligned}\int_0^t [O_3]dt &= \int_0^{t_b} [O_3]dt + \int_{t_b}^t [O_3]dt \\ &= \frac{[O_3]_0}{k_{dec.1}}(1-\exp(-k_{dec.1}t_b)) + \frac{[O_3]_{t_b}}{k_{dec.2}}(1-\exp(k_{dec.2}t_b - k_{dec.2}t)), \text{ if } t > t_b\end{aligned} \quad \text{(S8)}$$

For 2-cyclopenten-1-one and 2-ethynylbenzaldehyde, the k_{O_3} values at 25 °C are 4.7 × 10^3 and 2.2 × 10^2 $M^{-1}s^{-1}$, respectively (Table S4). At 5 °C they can be calculated using eq 2 in the main text, resulting in 1.9 × 10^3 and 5.6 × 10^1 $M^{-1}s^{-1}$, respectively. The abatement kinetics can be expressed as eq S1 in Text S4. Integrating the right half of eq S1 (Text S4), yields eq S9:

$$ln\frac{[C]}{[C]_0} = -k_{O_3}\int_0^t [O_3]dt \quad \text{(S9)}$$

Their relative residual concentration ($[C]/[C]_0$) can be calculated by eq S10 by considering of eqs S7 and S8.

$$\frac{[C]}{[C]_0} = \exp(-k_{O_3}\int_0^t [O_3]dt) \quad \text{(S10)}$$

According to a published study,[12] the kinetics for inactivation of *B. subtilis* spores exhibited a lag phase during which the inactivation efficiency is negligible followed by an inactivation phase during which pseudo-first kinetics are observed. The CTs for the lag phases (CT_{lag}) of inactivation of *B. subtilis* spores at 5 and 25 °C are 1.25 × 10^{-2} Ms and 2.5 × 10^{-3} Ms, respectively. The second-order inactivation rate constants ($k_{inact.}$) during the inactivation phase at 5 °C is 7.8 × 10^{2} $M^{-1}s^{-1}$,[12] and that at 25 °C was calculated to be 2.9 × 10^{3} $M^{-1}s^{-1}$ by the activation energy provided in the previous study.[12] Hence, the inactivation of *B. subtilis* spores (N/N_0) can be formulated by eqs S11 and S12.

$$\frac{N}{N_0} = 1, \text{ if } CT \leq CT_{lag} \quad \text{(S11)}$$

$$\frac{N}{N_0} = \exp(-k_{inact.}(CT - CT_{lag})), \text{ if } CT > CT_{lag} \quad \text{(S12)}$$

Text S13. Measurement of the second-order rate constants for reactions of ozone with *cis*- and *trans*-isomers of BDA

As shown in Figure 1, the synthesized BDA was present as a mixture of *cis*- and *trans*-isomers in the forms of hydrates. On average, the fractions of *cis*- and *trans*-BDA were 56% and 44%, respectively (Table S1). To obtain the second-order rate constants for the reactions of ozone with the *cis*- ($k_{O_3-cis\text{-BDA}}$) and *trans*-isomers of BDA ($k_{O_3-trans\text{-BDA}}$), two series of experiments (20 ± 1 °C, pH 2.3 ± 0.1) were conducted.

The experimental conditions and results for the 1st series are included in Table S4. In brief, 0.4 mM aqueous BDA solution was ozonated with ozone with a molar ratio of ≤ 10%. The decrease of ozone was measured by stopped-flow to obtain the k_{O_3} for the ozone reaction with the BDA mixture defined as $k_{O_3,\text{ overall-1}}$. The corresponding ozone kinetics can be expressed as follows (eq S13):

$$\frac{d[O_3]}{dt} = -(k_{O_3-cis\text{-BDA}}[cis\text{-BDA}] + k_{O_3-trans\text{-BDA}}[trans\text{-BDA}])[O_3] \quad \text{(S13)}$$

Introducing the fractions of *cis*- and *trans*-isomers to eq S13 yields eq S14.

$$k_{\mathrm{O_3,\,overall\text{-}1}} = 0.56k_{\mathrm{O_3}-cis\text{-BDA}} + 0.44k_{\mathrm{O_3}-trans\text{-BDA}} = (7.1 \pm 0.1)\times 10^{3}\ \mathrm{M^{-1}s^{-1}} \tag{S14}$$

The 2nd series of experiments included two steps: (1) A pre-ozonated BDA sample was prepared via ozonation of 0.7 mM BDA with 0.35 mM ozone, yielding a residual concentration of BDA (the mixture of *cis*- and *trans*-isomers) of 0.35 mM. Considering that $k_{\mathrm{O_3}-cis\text{-BDA}}$ and $k_{\mathrm{O_3}-trans\text{-BDA}}$ were likely different, the mole fractions of *cis*- and *trans*-isomers after pre-ozonation were changed. The residual concentrations of *cis*-BDA ([*cis*-BDA]$_{\mathrm{residual}}$) and *trans*-BDA ([*trans*-BDA]$_{\mathrm{residual}}$) after pre-ozonation can be calculated by eqs S15 and S16, respectively.

$$[cis\text{-BDA}]_{\mathrm{residual}} = (0.7\times 0.56 - 0.35\times\frac{0.56k_{\mathrm{O_3}-cis\text{-BDA}}}{0.56k_{\mathrm{O_3}-cis\text{-BDA}} + 0.44k_{\mathrm{O_3}-trans\text{-BDA}}})\ \mathrm{mM} \tag{S15}$$

$$[trans\text{-BDA}]_{\mathrm{residual}} = (0.7\times 0.44 - 0.35\times\frac{0.44k_{\mathrm{O_3}-trans\text{-BDA}}}{0.56k_{\mathrm{O_3}-cis\text{-BDA}} + 0.44k_{\mathrm{O_3}-trans\text{-BDA}}})\ \mathrm{mM} \tag{S16}$$

The fractions of residual *cis*-BDA and *trans*-BDA in pre-ozonated sample can then be calculated by eqs S17 and S18.

$$\begin{aligned}\text{fraction of } cis\text{-BDA} &= \alpha \\ &= \frac{[cis\text{-BDA}]_{\mathrm{residual}}}{[cis\text{-BDA}]_{\mathrm{residual}} + [trans\text{-BDA}]_{\mathrm{residual}}} \\ &= \frac{0.0672k_{\mathrm{O_3}-cis\text{-BDA}} + 0.4928k_{\mathrm{O_3}-trans\text{-BDA}}}{0.56k_{\mathrm{O_3}-cis\text{-BDA}} + 0.44k_{\mathrm{O_3}-trans\text{-BDA}}}\end{aligned} \tag{S17}$$

$$\begin{aligned}\text{fraction of } trans\text{-BDA} &= \beta \\ &= \frac{[trans\text{-BDA}]_{\mathrm{residual}}}{[cis\text{-BDA}]_{\mathrm{residual}} + [trans\text{-BDA}]_{\mathrm{residual}}} \\ &= \frac{0.4928k_{\mathrm{O_3}-cis\text{-BDA}} - 0.0528k_{\mathrm{O_3}-trans\text{-BDA}}}{0.56k_{\mathrm{O_3}-cis\text{-BDA}} + 0.44k_{\mathrm{O_3}-trans\text{-BDA}}}\end{aligned} \tag{S18}$$

(2) The pre-ozonated BDA solution obtained after step 1 was used as the BDA working solution, which was ozonated again and the ozone decrease was measured by stopped-flow. Similarly, $k_{\mathrm{O_3}}$ for ozone reaction with the pre-ozonated BDA sample is defined as $k_{\mathrm{O_3,\,overall\text{-}2}}$. The experimental conditions and results for stopped-flow spectrophotometric measurement are summarized as follows:

$[BDA]_{overall\text{-}2,\,0}$ (mM)	$[O_3]_0$ (mM)	$k_{O_3,\,overall\text{-}2}$ ($M^{-1}s^{-1}$)	average ± standard deviation of $k_{O_3,\,overall\text{-}2}$ ($M^{-1}s^{-1}$)
0.175	0.0175	4.1×10^3	
0.175	0.0175	4.0×10^3	$(4.1 \pm 0.1) \times 10^3$
0.175	0.015	4.1×10^3	

The ozone kinetics can be expressed as eq S19.

$$\frac{d[O_3]}{dt} = -(\alpha k_{O_3-cis\text{-BDA}} + \beta k_{O_3-trans\text{-BDA}})[O_3][BDA]_{overall\text{-}2} \quad (S19)$$

$k_{O_3,\,overall\text{-}2}$ can be expressed as follows (eq S20):

$$k_{O_3,\,overall\text{-}2} = \alpha k_{O_3-cis\text{-BDA}} + \beta k_{O_3-trans\text{-BDA}} = (4.1 \pm 0.1) \times 10^3 \ M^{-1}s^{-1} \quad (S20)$$

Via combination of eqs S14 and S20, $k_{O_3-cis\text{-BDA}}$ and $k_{O_3-trans\text{-BDA}}$ can be calculated and with two solutions:

(1) $k_{O_3-cis\text{-BDA}} = (3.0 \pm 0.04) \times 10^3\ M^{-1}s^{-1}$, $k_{O_3-trans\text{-BDA}} = (1.2 \pm 0.03) \times 10^4\ M^{-1}s^{-1}$;

(2) $k_{O_3-cis\text{-BDA}} = (1.1 \pm 0.02) \times 10^4\ M^{-1}s^{-1}$, $k_{O_3-trans\text{-BDA}} = (1.9 \pm 0.01) \times 10^3\ M^{-1}s^{-1}$.

Taking into account the potential *cis*-effect for BDA indicating that the *cis*-isomer is more stable than the corresponding *trans*-isomer,[13,14] the $k_{O_3-cis\text{-BDA}}$ should be lower than that of $k_{O_3-trans\text{-BDA}}$. Therefore, the k_{O_3} values for the reactions of ozone with *cis*- and *trans*-BDA hydrates are determined as $(3.0 \pm 0.04) \times 10^3$ and $(1.2 \pm 0.03) \times 10^4\ M^{-1}s^{-1}$, respectively.

Text S14. Identification of the transformation products (TP 150, TP 162, and TP 134) formed from ozonation of 2-ethynylbenzaldehyde

The common peak of m/z 105 in the negative mode (Figure S12a), which corresponds to m/z 107 in the positive mode (Figure S12b and c), represents the benzaldehyde fragment. The loss of 44 Da indicates an additional -COO group for TP 150 (Figure S12a). In the case of TP 134, the difference of 28 Da between the peak of m/z 107 and the molecular ion ($[M + H]^+$, m/z = 135) revealed the presence of a -CHO group (Figure S12b). The MS^2 spectra of TP 150 and TP 134 were virtually identical to

those of 2-carboxybenzaldehyde (Figure S12e) and phthaldialdehyde (Figure S12f), respectively. As shown in Figure S12c, the peaks of m/z 135 and m/z 163 lost 28 Da (-CO) and 56 Da (-CO-CO) to obtain the characteristic peak of the benzaldehyde fragment (m/z 107), respectively. Hence, TP 162 was proposed as a di-carbonyl compound.

Table S1. Fractions of *cis*- and *trans*-isomers of BDA hydrates for different rinsing cycles

batch	times of rinsing TSA	fractions of BDA isomers (%)	
		cis-isomer	*trans*-isomer
1	1	55.7	44.3
2	6	55.2	44.8
3	6	55.8	44.2
4	6	56.9	43.1
5	6	57.2	42.8

Table S2. Methods for the determination of second-order rate constants for the reactions of ozone with olefins and alkynes (groups I-V) at pH 2.3 or other indicated pH values

compound	p*K*a	method	pH (± 0.1)	T (°C)	WWL (nm) [d]	measured compound [e]	competitor
			**reference compound**				
cinnamic acid	4.44[15]	SF [b]	2.3	10–25	278	olefin	
			7.0	10–25	278	olefin	
			**group I: RHC=CHR**				
2-buten-1,4-dial$_{mix}$		SF	2.3	10–25	260	ozone	
cis-2-buten-1,4-diol		SF	2.3	10–25	260	ozone	
cis-1,4-dichloro-2-butene		SF	2.3	10–25	260	ozone	
fumaric acid	3.03, 4.44[16]	SF	2.3	10–25	283	ozone	
			3.0, 7.0	20 ± 1	220	olefin	
maleic acid	1.94, 6.22[17]	SF	2.3	10–25	278	ozone	
			1.7, 3.0, 6.0, 7.0, 7.8	20 ± 1	278	ozone	
			**group II: $R_1R_2C{=}CHR_3$**				
3-buten-1-ol		SF	2.3	10–25	260	ozone	
3-buten-2-ol		SF	2.3	10–25	260	ozone	
trans-2-methyl-2-butenal		SF	2.3	10–25	277	ozone	
trans-2-pentenal		SF	2.3	10–25	260	ozone	
		CK [c]	2.3	25 ± 2			cinnamic acid
			**group III: $R_1HC{=}CH{-}CH{=}CHR_2$**				
trans,*trans*-muconic acid	2.70, 4.66[18]	SF	2.3	10–25	270	olefin	
			3.0, 7.0	20 ± 1	270	olefin	
sorbic acid	4.76[19]	SF	2.3	10–25	272	olefin	
			7.0	20 ± 1	253	olefin	
sorbic alcohol		CK	2.3	25 ± 2			sorbic acid
sorbic aldehyde		SF	2.3	10–25	278	olefin	

**group IV: cyclic olefins**							
1-acetyl-1-cyclohexene		SF	2.3	10–25	279	ozone	
β-cyclocitral		CK	2.3	20 ± 2			*trans*-2-pentenal
2-cyclopenten-1-one		SF	2.3	10–25	260	ozone	
4-cyclopentene-1,3-dione		SF	2.3	10–25	260	ozone	
**group V: alkynes**							
3-butyn-1-ol		SF	2.3	10–25	260	ozone	
3-butynoic acid	3.62[20] [a]	SF	2.3	10–25	284	ozone	
			7.0	20 ± 1	284	ozone	
2-ethynylbenzaldehyde		SF	2.3	10–25	318	alkyne	
		CK	7.0	25 ± 2			bezafibrate

[a] Predicted value.

[b] SF: Stopped-flow method. The experiments were all performed in excess of olefins (or alkynes).

[c] CK: Competition kinetics.

[d] WWL: Working wavelength on stopped-flow spectrophotometer.

[e] Analyte measured by stopped-flow.

Table S3. Second-order rate constants ($k_{^{\bullet}OH}$) for the reactions of $^{\bullet}OH$ with selected olefins and alkynes and the corresponding reference compounds[21]

compound	$k_{^{\bullet}OH}$ ($M^{-1}s^{-1}$) [a]	reference compound	structure
cinnamic acid	8.1×10^9	cinnamic acid (anion)	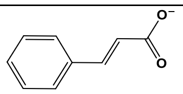
group I	6.0×10^9	fumaric acid (acid)	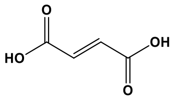
group II	5.8×10^9	crotonaldehyde	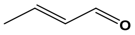
group III	7.0×10^9	butadiene	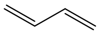
group IV	6.0×10^9	cyclopentene	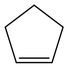
group V	4.7×10^9	acetylene	

[a] Due to the limited information on $k_{^{\bullet}OH}$ for the selected olefins and alkynes, their $k_{^{\bullet}OH}$ values were estimated by corresponding reference compounds with structural similarities. Hence, 100–130 molar excessive of *t*-BuOH to the selected compound was added to scavenge ≥ 90% $^{\bullet}OH$.

Table S4. Temperature dependence of second-order rate constants (k_{O_3}) for the reactions of ozone with selected olefins and alkynes (groups I-V) determined by stopped-flow in this study (experimental conditions are also shown)

compound	pH (± 0.1)	[phosphate buffer] (mM)	T (± 1, °C)	n [a]	[compound]$_0$ (mM)	[O_3]$_0$ (mM)	k_{O_3} ($M^{-1}s^{-1}$)	ave. k_{O_3} ± error ($M^{-1}s^{-1}$) [b]
**reference compound**								
cinnamic acid	2.3	10	10	2	0.075	0.0075	5.3×10^4	$(5.2 \pm 0.1) \times 10^4$
					0.075	0.005	5.1×10^4	
			15	2	0.075	0.0075	6.1×10^4	$(6.2 \pm 0.1) \times 10^4$
					0.075	0.005	6.3×10^4	
			20	5	0.075	0.0075	7.7×10^4	$(7.5 \pm 0.2) \times 10^4$
					0.075	0.0075	7.4×10^4	
					0.075	0.0075	7.3×10^4	
					0.075	0.005	7.7×10^4	
					0.075	0.005	7.4×10^4	
			25	6	0.075	0.0075	9.6×10^4	$(9.0 \pm 0.8) \times 10^4$
					0.075	0.0075	8.3×10^4	
					0.075	0.0075	8.4×10^4	
					0.075	0.005	1.0×10^5	
					0.075	0.005	8.4×10^4	
					0.075	0.005	9.2×10^4	
	7.0	10	10	4	0.075	0.0075	6.7×10^5	$(6.6 \pm 0.5) \times 10^5$
					0.075	0.0075	6.4×10^5	
					0.075	0.00625	7.2×10^5	
					0.075	0.00625	6.1×10^5	
			15	2	0.075	0.0075	7.5×10^5	$(7.6 \pm 0.2) \times 10^5$
					0.075	0.00625	7.7×10^5	
			20	3	0.075	0.0075	9.0×10^5	$(8.9 \pm 0.2) \times 10^5$

					0.075	0.00625	9.1×10^5	
					0.075	0.00625	8.6×10^5	
			25	3	0.075	0.0075	1.0×10^6	$(1.0 \pm 0.02) \times 10^6$
					0.075	0.00625	1.0×10^6	
					0.075	0.005	1.0×10^6	
**group I: RHC=CHR**								
2-buten-1,4-dial$_{mix}$	2.3	10	10	3	0.4	0.04	4.8×10^3	$(4.7 \pm 0.1) \times 10^3$
					0.4	0.04	4.6×10^3	
					0.4	0.03	4.6×10^3	
			15	2	0.4	0.04	5.5×10^3	$(5.5 \pm 0.1) \times 10^3$
					0.4	0.03	5.6×10^3	
			20	2	0.4	0.04	7.0×10^3	$(7.1 \pm 0.1) \times 10^3$
					0.4	0.03	7.2×10^3	
			25	2	0.4	0.04	8.5×10^3	$(8.7 \pm 0.2) \times 10^3$
					0.4	0.03	8.9×10^3	
cis-2-buten-1,4-diol	2.3	10	10	2	0.5	0.05	2.7×10^5	$(2.7 \pm 0.04) \times 10^5$
					0.5	0.0375	2.7×10^5	
			15	2	0.5	0.05	3.0×10^5	$(3.0 \pm 0.1) \times 10^5$
					0.5	0.0375	3.1×10^5	
			20	4	0.5	0.05	3.4×10^5	$(3.4 \pm 0.1) \times 10^5$
					0.5	0.05	3.4×10^5	
					0.5	0.0375	3.6×10^5	
					0.5	0.0375	3.4×10^5	
			25	4	0.5	0.05	3.9×10^5	$(3.9 \pm 0.01) \times 10^5$
					0.5	0.0375	3.9×10^5	
cis-1,4-dichloro-2-butene	2.3	10	10	2	0.5	0.05	1.2×10^4	$(1.2 \pm 0.02) \times 10^4$
					0.5	0.0375	1.2×10^4	
			15	2	0.5	0.05	1.6×10^4	$(1.6 \pm 0.03) \times 10^4$

					0.5	0.0375	1.6×10^4	
			20	3	0.5	0.05	1.9×10^4	$(1.9 \pm 0.03) \times 10^4$
					0.5	0.05	1.9×10^4	
					0.5	0.0375	2.0×10^4	
			25	2	0.5	0.05	2.4×10^4	$(2.4 \pm 0.04) \times 10^4$
					0.5	0.0375	2.3×10^4	
fumaric acid	2.3	100	10	4	1.5	0.15	5.2×10^3	$(5.2 \pm 0.01) \times 10^3$
					1.5	0.15	5.2×10^3	
					1.5	0.125	5.2×10^3	
					1.5	0.125	5.1×10^3	
			15	4	1.5	0.15	6.1×10^3	$(6.2 \pm 0.1) \times 10^3$
					1.5	0.15	6.2×10^3	
					1.5	0.125	6.2×10^3	
					1.5	0.125	6.2×10^3	
			20	2	1.5	0.15	7.6×10^3	$(7.6 \pm 0.1) \times 10^3$
					1.5	0.125	7.7×10^3	
			25	2	1.5	0.15	9.2×10^3	$(9.2 \pm 0.04) \times 10^3$
					1.5	0.125	9.2×10^3	
	7.0	100	20	2	0.1	0.01	1.3×10^5	$(1.3 \pm 0.01) \times 10^5$
					0.1	0.009	1.3×10^5	
	3.0	100	20	2	0.1	0.01	1.5×10^4	$(1.5 \pm 0.02) \times 10^4$
					0.1	0.009	1.5×10^4	
maleic acid	2.3	10	10	2	4.25	0.425	1.0×10^3	$(1.1 \pm 0.02) \times 10^3$
					4.25	0.2	1.1×10^3	
			15	2	4.25	0.425	1.3×10^3	$(1.3 \pm 0.03) \times 10^3$
					4.25	0.2	1.3×10^3	
			20	4	4.25	0.425	1.6×10^3	$(1.6 \pm 0.1) \times 10^3$
					4.25	0.2	1.7×10^3	
					2.0	0.2	1.5×10^3	

					2.0	0.1	1.5×10^3	
			25	2	2.0	0.2	2.0×10^3	$(2.0 \pm 0.1) \times 10^3$
					2.0	0.1	2.1×10^3	
	7.0	20	20	2	0.5	0.05	4.9×10^3	$(4.9 \pm 0.01) \times 10^3$
					0.5	0.04	4.9×10^3	
	1.7	100	20	2	0.5	0.05	9.7×10^2	$(9.7 \pm 0.1) \times 10^2$
					0.5	0.04	9.8×10^2	
	3.0	20	20	2	0.5	0.05	2.2×10^3	$(2.2 \pm 0.02) \times 10^3$
					0.5	0.04	2.2×10^3	
	6.0	50	20	2	0.5	0.05	3.5×10^3	$(3.7 \pm 0.4) \times 10^3$
					0.5	0.04	4.0×10^3	
	7.8	50	20	2	0.5	0.05	5.1×10^3	$(5.2 \pm 0.2) \times 10^3$
					0.5	0.04	5.3×10^3	
**group II: $R_1R_2C{=}CHR_3$**								
3-buten-1-ol	2.3	10	10	3	0.5	0.05	2.6×10^5	$(2.6 \pm 0.02) \times 10^5$
					0.5	0.04	2.6×10^5	
					0.5	0.04	2.6×10^5	
			15	2	0.5	0.05	3.0×10^5	$(3.0 \pm 0.1) \times 10^5$
					0.5	0.04	3.1×10^5	
			20	2	0.5	0.05	3.5×10^5	$(3.6 \pm 0.1) \times 10^5$
					0.5	0.04	3.6×10^5	
			25	3	0.5	0.05	4.2×10^5	$(4.2 \pm 0.04) \times 10^5$
					0.5	0.05	4.2×10^5	
					0.5	0.04	4.2×10^5	
3-buten-2-ol	2.3	10	10	2	0.5	0.05	7.0×10^4	$(7.0 \pm 0.04) \times 10^4$
					0.5	0.04	7.1×10^4	
			15	2	0.5	0.05	8.3×10^4	$(8.4 \pm 0.04) \times 10^4$
					0.5	0.04	8.4×10^4	

			20	2	0.5	0.05	9.9×10^4	$(9.9 \pm 0.04) \times 10^4$
					0.5	0.04	9.9×10^4	
			25	2	0.5	0.05	1.1×10^5	$(1.2 \pm 0.01) \times 10^5$
					0.5	0.04	1.2×10^5	
trans-2-methyl-2-butenal	2.3	10	10	3	1.5	0.15	2.7×10^4	$(2.8 \pm 0.04) \times 10^4$
					1.5	0.125	2.8×10^4	
					1.5	0.125	2.8×10^4	
			15	2	1.5	0.15	3.3×10^4	$(3.3 \pm 0.1) \times 10^4$
					1.5	0.125	3.4×10^4	
			20	2	1.5	0.15	4.2×10^4	$(4.2 \pm 0.01) \times 10^4$
					1.5	0.125	4.2×10^4	
			25	3	1.5	0.15	5.2×10^4	$(5.2 \pm 0.04) \times 10^4$
					1.5	0.15	5.2×10^4	
					1.5	0.125	5.3×10^4	
trans-2-pentenal	2.3	10	10	3	1.0	0.075	6.0×10^3	$(5.8 \pm 0.2) \times 10^3$
					0.5	0.05	5.6×10^3	
					0.5	0.0375	6.0×10^3	
			15	6	1.0	0.075	7.7×10^3	$(7.4 \pm 0.7) \times 10^3$
					1.0	0.075	7.5×10^3	
					0.5	0.05	6.4×10^3	
					0.5	0.05	6.7×10^3	
					0.5	0.0375	8.4×10^3	
					0.5	0.0375	7.5×10^3	
			20	5	1.0	0.075	1.0×10^4	$(1.0 \pm 0.1) \times 10^4$
					0.5	0.05	9.6×10^3	
					0.5	0.05	9.5×10^3	
					0.5	0.0375	1.2×10^4	
					0.5	0.0375	1.1×10^4	
			25	6	1.0	0.075	1.3×10^4	$(1.2 \pm 0.1) \times 10^4$

					1.0	0.075	1.3 × 10^4	
					0.5	0.05	1.1 × 10^4	
					0.5	0.05	1.3 × 10^4	
					0.5	0.05	1.0 × 10^4	
					0.5	0.0375	1.4 × 10^4	
**group III: $R_1HC=CH-CH=CHR_2$**								
trans,*trans*-muconic acid	2.3	10	10	2	0.1	0.01	6.2 × 10^3	(6.0 ± 0.4) × 10^3
					0.1	0.0075	5.7 × 10^3	
			15	2	0.1	0.01	7.2 × 10^3	(7.4 ± 0.2) × 10^3
					0.1	0.0075	7.5 × 10^3	
			20	2	0.1	0.01	8.2 × 10^3	(8.3 ± 0.2) × 10^3
					0.1	0.0075	8.5 × 10^3	
			25	2	0.1	0.01	1.0 × 10^4	(1.0 ± 0.03) × 10^4
					0.1	0.0075	9.8 × 10^3	
	7.0	10	20	2	0.05	0.005	1.7 × 10^5	(1.7 ± 0.01) × 10^5
					0.05	0.004	1.7 × 10^5	
	3.0	10	20	3	0.1	0.01	1.3 × 10^4	(1.2 ± 0.1) × 10^4
					0.1	0.01	1.3 × 10^4	
					0.1	0.0075	1.1 × 10^4	
sorbic acid	2.3	10	10	2	0.05	0.005	2.9 × 10^5	(3.0 ± 0.1) × 10^5
					0.05	0.0025	3.0 × 10^5	
			15	2	0.05	0.005	3.5 × 10^5	(3.7 ± 0.3) × 10^5
					0.05	0.0025	3.9 × 10^5	
			20	2	0.05	0.005	4.3 × 10^5	(4.4 ± 0.1) × 10^5
					0.05	0.0025	4.5 × 10^5	
			25	2	0.05	0.005	5.2 × 10^5	(5.2 ± 0.04) × 10^5
					0.05	0.0025	5.1 × 10^5	
	7.0	10	20	3	0.05	0.005	2.0 × 10^6	(2.0 ± 0.1) × 10^6

					0.05	0.004	2.0×10^6	
					0.075	0.0075	1.9×10^6	
sorbic aldehyde	2.3	10	10	2	0.05	0.005	1.1×10^5	$(1.1 \pm 0.04) \times 10^5$
					0.05	0.0025	1.1×10^5	
			15	2	0.05	0.005	1.4×10^5	$(1.4 \pm 0.01) \times 10^5$
					0.05	0.0025	1.4×10^5	
			20	2	0.05	0.005	1.8×10^5	$(1.8 \pm 0.04) \times 10^5$
					0.05	0.0025	1.8×10^5	
			25	2	0.05	0.005	2.2×10^5	$(2.2 \pm 0.1) \times 10^5$
					0.05	0.0025	2.2×10^5	
**group IV: cyclic olefins**								
1-acetyl-1-cyclohexene	2.3	10	10	2	1.0	0.1	1.4×10^5	$(1.4 \pm 0.02) \times 10^5$
					1.0	0.0875	1.4×10^5	
			15	2	1.0	0.1	1.6×10^5	$(1.6 \pm 0.03) \times 10^5$
					1.0	0.0875	1.6×10^5	
			20	3	1.0	0.1	2.0×10^5	$(2.0 \pm 0.01) \times 10^5$
					1.0	0.0875	2.0×10^5	
					1.0	0.05	2.0×10^5	
			25	4	1.0	0.1	2.3×10^5	$(2.3 \pm 0.04) \times 10^5$
					1.0	0.1	2.2×10^5	
					1.0	0.0875	2.3×10^5	
					1.0	0.0875	2.3×10^5	
2-cyclopenten-1-one	2.3	10	10	2	1.5	0.075	2.3×10^3	$(2.3 \pm 0.1) \times 10^3$
					1.0	0.1	2.3×10^3	
			15	2	1.5	0.075	3.4×10^3	$(3.4 \pm 0.1) \times 10^3$
					1.0	0.1	3.3×10^3	
			20	2	1.5	0.075	4.2×10^3	$(4.2 \pm 0.1) \times 10^3$
					1.0	0.1	4.1×10^3	

			25	4	1.5	0.075	4.7×10^3	$(4.7 \pm 0.2) \times 10^3$
					1.5	0.075	4.8×10^3	
					1.0	0.1	4.4×10^3	
					1.0	0.05	4.7×10^3	
4-cyclopentene-1,3-dione	2.3	10	10	2	2.85	0.285	1.3×10^2	$(1.3 \pm 0.04) \times 10^2$
					2.85	0.15	1.3×10^2	
			15	2	2.85	0.285	1.8×10^2	$(1.8 \pm 0.01) \times 10^2$
					2.85	0.15	1.8×10^2	
			20	2	2.85	0.285	2.2×10^2	$(2.3 \pm 0.1) \times 10^2$
					2.85	0.15	2.4×10^2	
			25	2	2.85	0.285	2.9×10^2	$(3.0 \pm 0.02) \times 10^2$
					2.85	0.15	3.0×10^2	
**group V: alkynes**								
3-butyn-1-ol	2.3	10	10	2	4.5	0.375	2.8×10^2	$(2.9 \pm 0.1) \times 10^2$
					4.5	0.25	2.9×10^2	
			15	2	4.5	0.375	3.8×10^2	$(3.8 \pm 0.1) \times 10^2$
					4.5	0.25	3.7×10^2	
			20	2	4.5	0.375	4.8×10^2	$(4.9 \pm 0.2) \times 10^2$
					4.5	0.25	5.0×10^2	
			25	2	4.5	0.375	6.2×10^2	$(6.3 \pm 0.2) \times 10^2$
					4.5	0.25	6.4×10^2	
3-butynoic acid	2.3	100	10	2	2.0	0.2	1.1×10^2	$(1.1 \pm 0.02) \times 10^2$
					2.0	0.175	1.1×10^2	
			15	2	2.0	0.2	1.4×10^2	$(1.4 \pm 0.01) \times 10^2$
					2.0	0.175	1.5×10^2	
			20	2	2.0	0.2	1.9×10^2	$(2.0 \pm 0.1) \times 10^2$
					2.0	0.175	2.0×10^2	
			25	2	2.0	0.2	2.7×10^2	$(2.7 \pm 0.03) \times 10^2$

					2.0	0.175	2.7×10^2	
	7.0	100	20	3	1.0	0.1	6.0×10^2	$(6.2 \pm 0.4) \times 10^2$
					1.0	0.0875	5.9×10^2	
					1.0	0.0875	6.7×10^2	
2-ethynylbenzaldehyde	2.3	10	10	2	0.475	0.0375	7.6×10^1	$(8.0 \pm 0.6) \times 10^1$
					0.475	0.03	8.5×10^1	
			15	2	0.475	0.0375	1.1×10^2	$(1.1 \pm 0.1) \times 10^2$
					0.475	0.03	1.2×10^2	
			20	2	0.475	0.0375	1.6×10^2	$(1.6 \pm 0.03) \times 10^2$
					0.475	0.03	1.6×10^2	
			25	2	0.475	0.0375	2.2×10^2	$(2.2 \pm 0.01) \times 10^2$
					0.475	0.03	2.2×10^2	

[a] Number of repetitions.

[b] Ave. k_{O_3} is the mean k_{O_3} value; ± error indicates the deviation of replicated measurements from the mean. The values determined at 20 ± 1 °C are also included in Table 1.

Table S5. Summary of previously reported second-order rate constants (k_{O_3}) and activation energies (E_a) for the reactions of ozone with olefins and alkynes

compound	structure	species-specific second-order rate constant ($M^{-1}s^{-1}$)			k_{O_3} ($M^{-1}s^{-1}$)	pH	T (°C)	E_a (kJ mol^{-1})	ref.
		neutral	monoanion	dianion					
**olefins**									
acrylamide	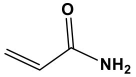				1.0×10^5	5.4–5.8	22 ± 1		22
acrylic acid	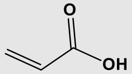	2.8×10^4	1.6×10^5						23
acrylonitrile	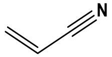				6.7×10^2				24
allylbenzene	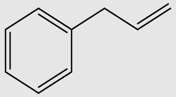				1.2×10^5	2	23 ± 2		25
1,4-benzoquinone	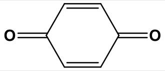				2.5×10^3	7, 10			26
3-buten-2-ol	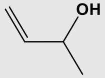				7.9×10^4<br>9.1×10^4		 21 ± 1		27<br>28
β-caryophyllonic acid	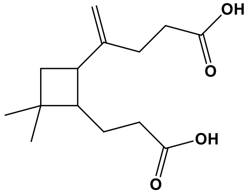				4.8×10^5<br>6.0×10^5	2<br>8	22 ± 2<br>22 ± 2		29<br>29
chlordane	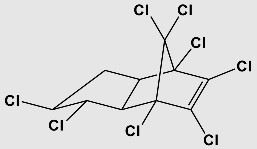				$\leq 4\times 10^{-2}$	6.2–6.4	19 ± 1		22

				7.6 × 10^5	6.5	22	21.2	30
				3.1 × 10^4	2			31
cinnamic acid	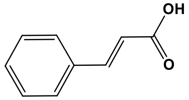	5.8 × 10^4	7.5 × 10^5	7.8 × 10^5	7.2	20 ± 1	19.1	32
		5.0 × 10^4	3.8 × 10^5			20–21		33
		1.0 × 10^5	1.2 × 10^6					34
β-cyclocitral	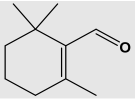			3.9 × 10^3	2	20–25		35
cyclohexene	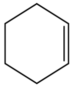			2.2 × 10^6		25		36
1,2-dibromoethene	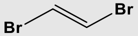			1.5 × 10^3				24
1,1-dichloroethene	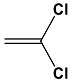			1.1 × 10^2	2	20 ± 0.5		25
				5.4 × 10^2			34	27
cis-1,2-dichloroethene	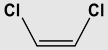			< 8.0 × 10^2	2	20 ± 0.5		25
				3.1 × 10^2	2	25		22
trans-1,2-dichloroethene	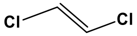			6.5 × 10^3			31	27
				5.7 × 10^3	2.0	20 ± 0.5		25
dichloromaleic acid	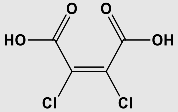			1.0 × 10^1	3.3			23
1,1-dichloropropene	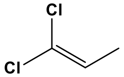			2.6 × 10^3				27
diethyl vinyl phosphonate	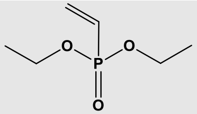			3.3 × 10^3				24
3,4-dihydroxycinnamic acid	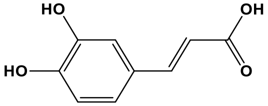	2.0 × 10^6	1.2 × 10^7					34

endrin	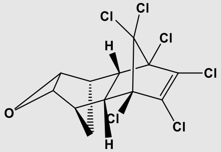				< 2.0 × 10^{-2}	2.7–6.4	23 ± 3		22
ethene					1.8 × 10^{5}				27
fumaric acid	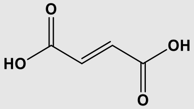				6.0 × 10^{3}	2	23 ± 2		37
					1.0 × 10^{5}	5	23 ± 2		37
					1.5 × 10^{5}	7			38
		8.5 × 10^{3}		6.5 × 10^{4}					23
hexachloro-1,3-butadiene	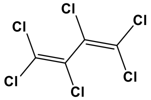				< 1.0 × 10^{-1}	2			39
hexachlorocyclopentadiene	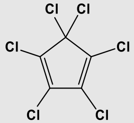				9 × 10^{1}	2.7–6.3	25 ± 2		22
3-hexen-1-ol	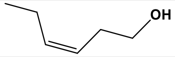				5.4 × 10^{5}	2	20–25		35
1-hexene-3-ol	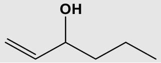				1.0 × 10^{5}	2	20 ± 0.5		25
1-hexene-4-ol	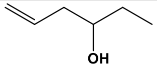				1.8 × 10^{5}	2	20 ± 0.5		25
2-hexenoic acid	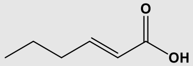		3.4 × 10^{5}				22–24		40
β-ionone	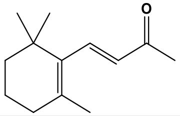				1.6 × 10^{5}		20–25		35
isoprene	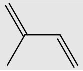				3.7 × 10^{5}	2	20	19.9	41
limonic acid	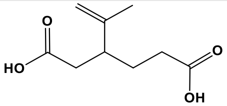	2.3 × 10^{4}	4.6 × 10^{4}						31

limononic acid	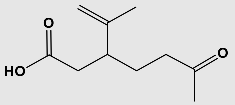	2.4×10^4	6.1×10^4				25 ± 2		31
		2.6×10^4	5.7×10^4				25 ± 2		31
					4.2×10^4	2	25 ± 2		42
maleic acid	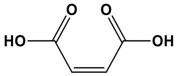				1.0×10^3	2	23 ± 2		37
					5.0×10^3	6	23 ± 2		37
					1.2×10^4	7	20		43
		1.4×10^3	4.2×10^3	7×10^3					23
				2.4×10^4			22–24		40
methacrolein	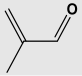				1.9×10^4	2	20	23.9	41
methacrylic acid	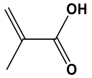	1.5×10^5	3.7×10^6						23
4-methoxy cinnamic acid	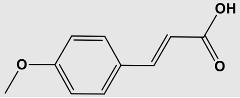	1.3×10^5	6.8×10^5				20–21		33
3-methoy-4-hydroxy cinnamic acid	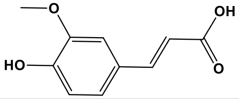	1.1×10^6	7.9×10^6						34
methyl vinyl ketone	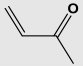				3.7×10^4	2	20	18.0	41
cis,*cis*-muconic acid	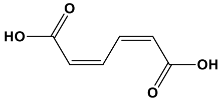				2.6×10^4	3.1			23
cis,*trans*-muconic acid	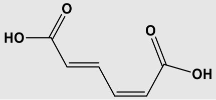				1.4×10^4	3	21–23		18
					2.5×10^5	7	21–23		18
trans,*trans*-muconic acid	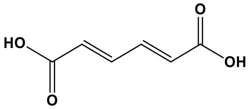				1.6×10^4	3	20		44
					1.4×10^5	7	20		44
					1.5×10^4	3	21–23		18
					1.3×10^5	7	21–23		18

4-nitrocinnamic acid	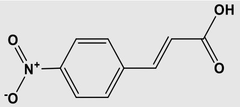	1.2×10^{5}			20–21	33
2,6-nonadienal	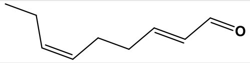		8.7×10^{5}	2	20–25	35
(*Z*)-1,1,2,3,4-pentachloro-1,3-butadiene	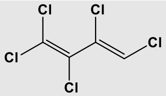		8.0×10^{-1}	2		39
1,1,2,4,4-pentachloro-1,3-butadiene	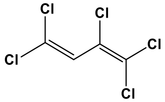		1.0×10^{1}	2		39
1-penten-3-one	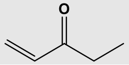		5.9×10^{4}	2	20–25	35
phenyl vinylsulfonate	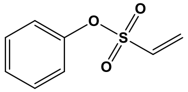		2.0×10^{2}			24
propene	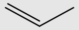		8.0×10^{5}			27
sorbic acid	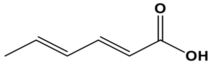		3.2×10^{5}	3		45
			9.6×10^{5}	8		45
styrene	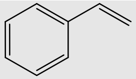		3.0×10^{5}	2	23 ± 2	25
α-terpineol	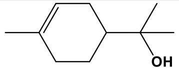		9.9×10^{6}			46
1,1,2,3-tetrachloro-1,3-butadiene	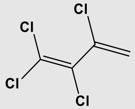		3.0×10^{2}	2		39
(*E*)-1,1,2,4-tetrachloro-1,3-butadiene	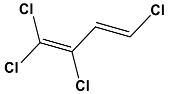		7.8×10^{3}	2		39
(*E*)-1,1,3,4-tetrachloro-1,3-butadiene	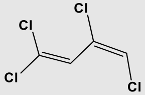		1.1×10^{3}	2		39

(*Z*)-1,1,3,4-tetrachloro-1,3-butadiene	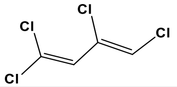				4.0×10^{2}	2			39
1,1,4,4-tetrachloro-1,3-butadiene	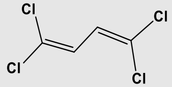				2.7×10^{2}	2			39
(*Z,Z*)-1,2,3,4-tetrachloro-1,3-butadiene	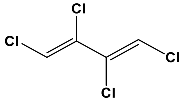				1.6×10^{2}	2			39
tetrachloroethene	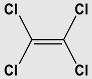				$< 1.0 \times 10^{-1}$	2	20 ± 0.5		25
tetramethylethene	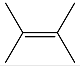				$> 1.0 \times 10^{6}$				27
trichloroethene	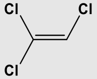				1.4×10^{1}			35.1 [a]	27
					1.7×10^{1}	2	20 ± 0.5		25
					1.5×10^{1}	2	21 ± 1		22
					1.7×10^{1}				47
vinyl acetate	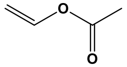				1.6×10^{5}				24
vinyl bromide	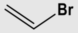				1.0×10^{4}				24
vinyl chloride	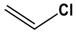				1.4×10^{4}				27
vinylene carbonate	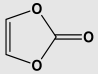				2.6×10^{4}				24
vinyl phosphonic acid	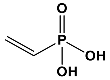	1.0×10^{4}	2.7×10^{4}	1.0×10^{5}					24
vinyl sulfonic acid	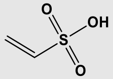		8.3×10^{3}						24
**alkynes**									
1-ethinyl-1-cyclohexanol	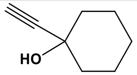				2×10^{2}	8	20		48

[a] Pryor et al (1984) reported an E_a value from a previous study which is not publicly available.

Table S6. Second-order rate constants (k_{O_3}) for the reactions of ozone with cyclic olefins and the corresponding non-cyclic analogues

cyclic olefin	chemical structure	$k_{O_3,\text{ cyclic olefin}}$ ($M^{-1}s^{-1}$)	non-cyclic olefin	chemical structure	$k_{O_3,\text{ model olefin}}$ ($M^{-1}s^{-1}$)
cyclohexene	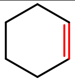	2.2×10^6 [36]	ethene		1.8×10^5 [27]
1-acetyl-1-cyclohexene	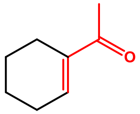	$(2.0 \pm 0.01) \times 10^5$ (this study)	methyl vinyl ketone	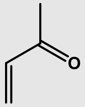	3.7×10^4 [41]
β-cyclocitral	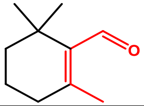	$(6.8 \pm 0.4) \times 10^3$ (this study)<br>3.9×10^3 [35]	*trans*-2-methyl-2-butenal [a]	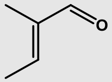	$(4.2 \pm 0.01) \times 10^4$ (this study)

[a] Due to the lack of k_{O_3} for the reaction with ozone and structural similarity with 2-butenal, *trans*-2-methyl-2-butenal was selected as the non-cyclic analogue for *β*-cyclocitral.

Table S7. Examples for reported second-order rate constants (k_{O_3}) for the reactions of ozone with heterocycles with an olefinic moiety as the dominant reactive site

base heterocycle	compound	structure	k_{O_3} ($M^{-1}s^{-1}$)	pH	T (°C)	ref.
		**N-containing heterocycles**				
imidazole	imidazole (protonated)	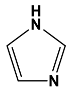	2.2×10^1		23 ± 2	37
			2.1×10^2		23 ± 0.1	40
			1.5×10^3			49
	4-methylimidazole (protonated)	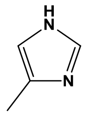	1.7×10^3		23 ± 0.1	40
pyrrole	maleimide	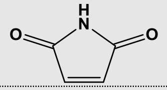	4.2×10^3	7.0		49
	pyrrole	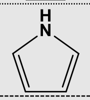	8.6×10^5	7.0		49
		**O-containing heterocycles**				
furan	furan-2,5-dicarboxylic acid	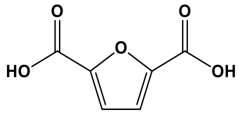	8.5×10^4	7.0		50
	2-furoic acid	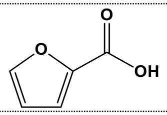	5.9×10^5	7.0		50
	3-(2-furyl) propanoic acid	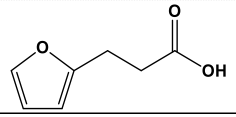	3.2×10^6	7.0		50

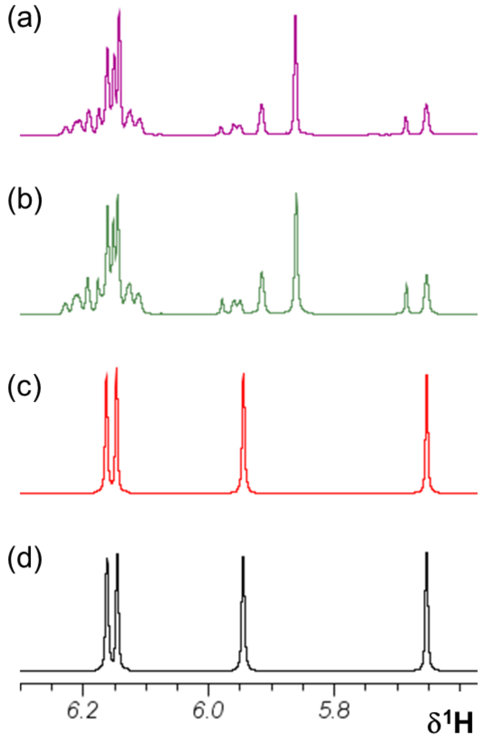

Figure S1. Regions of interest of the ^{1}H NMR spectra of (a) 1 M DMDF hydrolyzed for two weeks, (b) 1.2 M DMDF hydrolyzed for several minutes in the presence of 0.16 M DCl, (c) 1.2 M DMDF in the presence of 0.20 M NaOD, and (d) 1.2 M DMDF (H_2O:D_2O = 9:1).

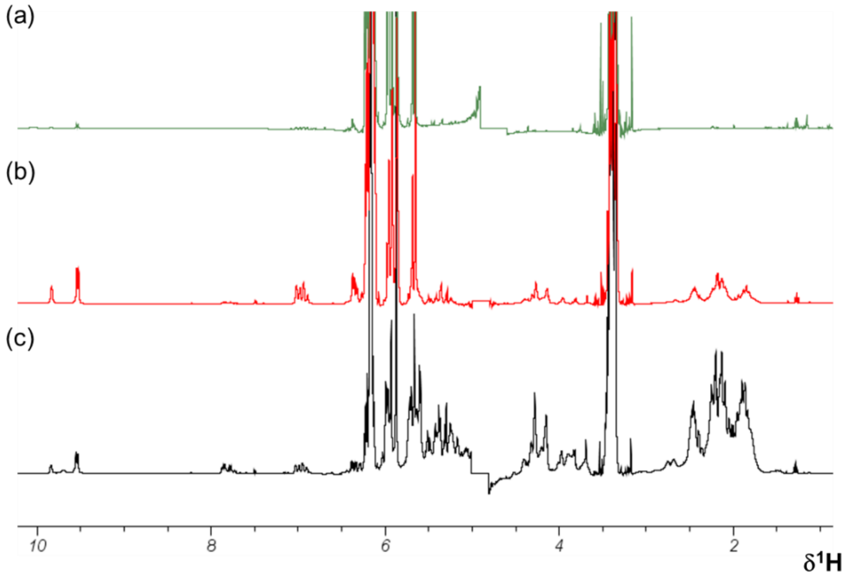

Figure S2. ^{1}H NMR spectra of a 1.2 M DMDF solution hydrolyzed in the presence of 0.16 M DCl recorded after (a) several minutes, and (b) 12 h (additional application of a weak air stream to remove methanol for approximately 1 h), and (c) 3 days. The resonances of the BDA main product of the three spectra are normalized to the same intensities.

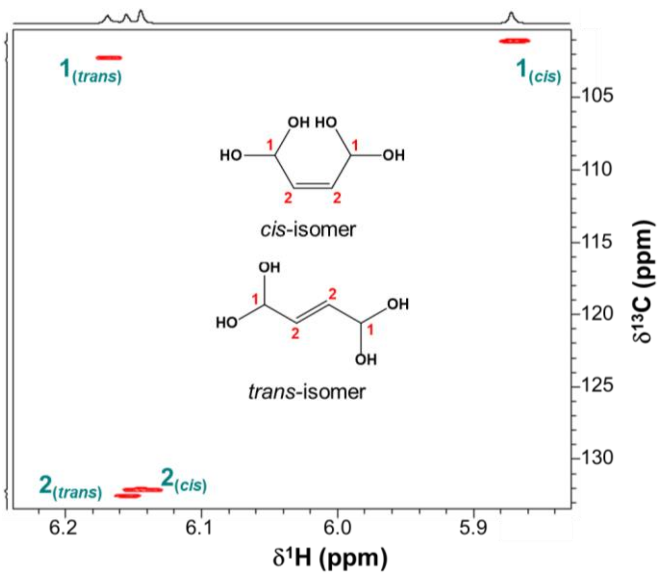

Figure S3. Characterization of BDA species by NMR spectroscopy: Region of interest of ^{1}H-^{13}C HSQC NMR spectrum with chemical structures and resonance assignments of a 1 mM BDA solution (H_2O:D_2O = 9:1).

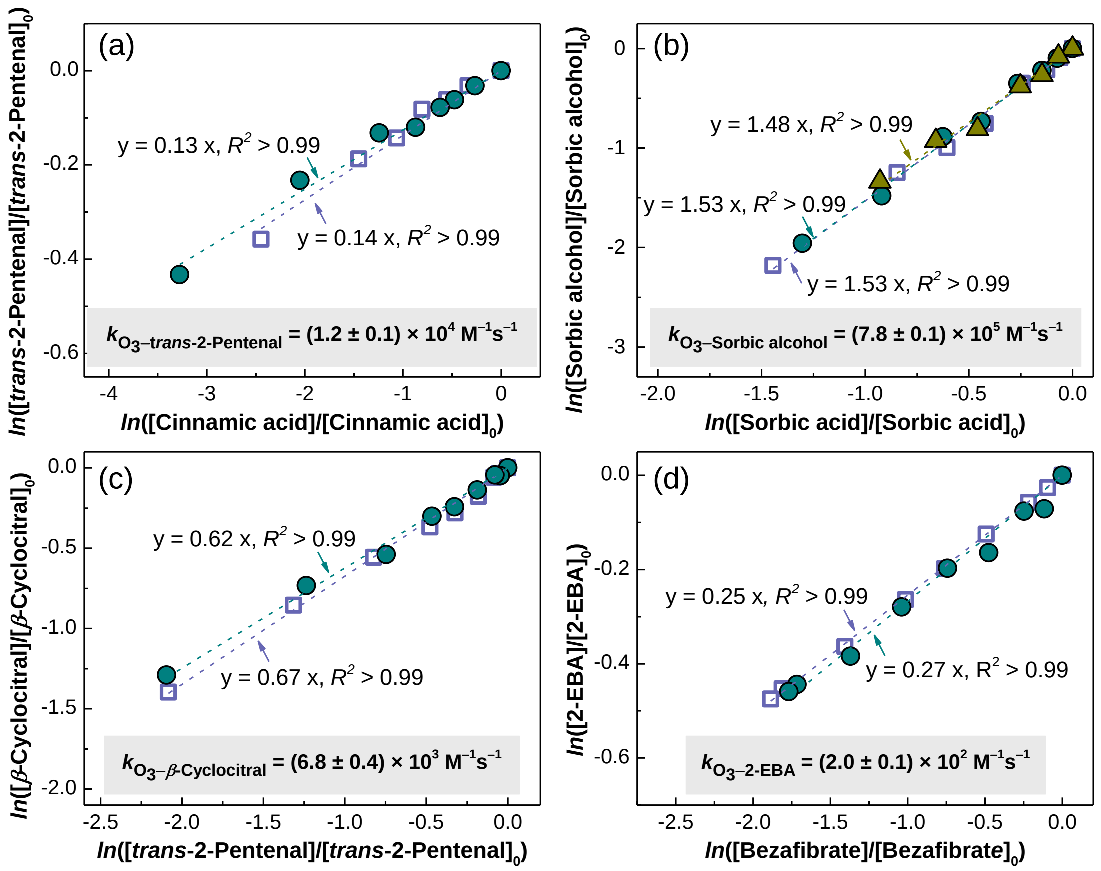

Figure S4. Competition kinetics plots for the determination of second-order rate constants (k_{O_3}) for the reactions of ozone with (a) *trans*-2-pentenal using cinnamic acid as a competitor (n = 2), (b) sorbic alcohol using sorbic acid as a competitor (n = 3), (c) *β*-cyclocitral using *trans*-2-pentenal as a competitor (n = 2), and (d) 2-ethynylbenzaldehyde (2-EBA) using bezafibrate as a competitor (n = 2). The values of "n" represent the number of repetitions. Dashed lines represent linear regressions, with the ratio of k_{O_3} for the target compound ($k_{O_3\text{-C}}$) and k_{O_3} for the corresponding reference compound ($k_{O_3\text{-R}}$) as the slope (see eq 3 in the main text). Experimental conditions: (a) [cinnamic acid]$_0$ = 150 μM, [*trans*-2-pentenal]$_0$ = 300 μM, $[O_3]_0$ = 0–250 μM, [*t*-BuOH]$_0$ = 0.1 M, pH 2.3 ± 0.1, 10 mM phosphate buffer, T = 25 ± 2 °C; (b) [sorbic acid]$_0$ = 100 μM, [sorbic alcohol]$_0$ = 75 μM, $[O_3]_0$ = 0–165 μM, [*t*-BuOH]$_0$ = 0.1 M, pH 2.3 ± 0.1, 10 mM phosphate buffer, T = 25 ± 2 °C; (c) [*trans*-2-pentenal]$_0$ = 150 μM, [*β*-cyclocitral]$_0$ = 300 μM, $[O_3]_0$ = 0–314 μM, [*t*-BuOH]$_0$ = 0.4 M, pH = 2.3 ± 0.1, 10 mM phosphate buffer, T = 20 ± 2 °C; and (d) [bezafibrate]$_0$ = 30 μM, [2-EBA]$_0$ = 50 μM, $[O_3]_0$ = 0–72 μM, [*t*-BuOH]$_0$ = 0.1 M, pH 7.0 ± 0.1, 10 mM phosphate buffer, T = 25 ± 2 °C. To be noted, due to the limited solubility of *β*-cyclocitral and bezafibrate,

their stock solutions were prepared in a mixture of *t*-BuOH and ultra-purified water, which resulted in *t*-BuOH concentrations of 0.4 M and 0.1 M, respectively. k_{O_3} (pH 7.0, 25 °C) for bezafibrate is calculated to be 772 $M^{-1}s^{-1}$ using eq 2 in the main text from the reported k_{O_3} (pH 7.0, 20 °C) of 590 $M^{-1}s^{-1}$ and an activation energy of 39 kJ mol^{-1}.[51]

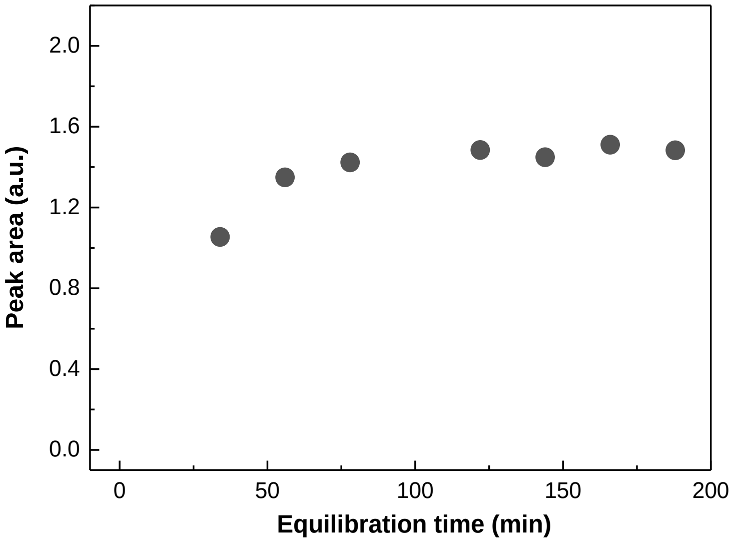

Figure S5. Effect of equilibration time for an aqueous glyoxal stock solution on the signal intensity (HPLC-UV (305 nm)) of glyoxal hydrazone. A 0.84 mM glyoxal stock solution in ultra-purified water was first prepared and equilibrated for different durations, then a 42 µM glyoxal aqueous solution was derivatized following the procedure outlined in Text S5 and then determined by HPLC-UV.

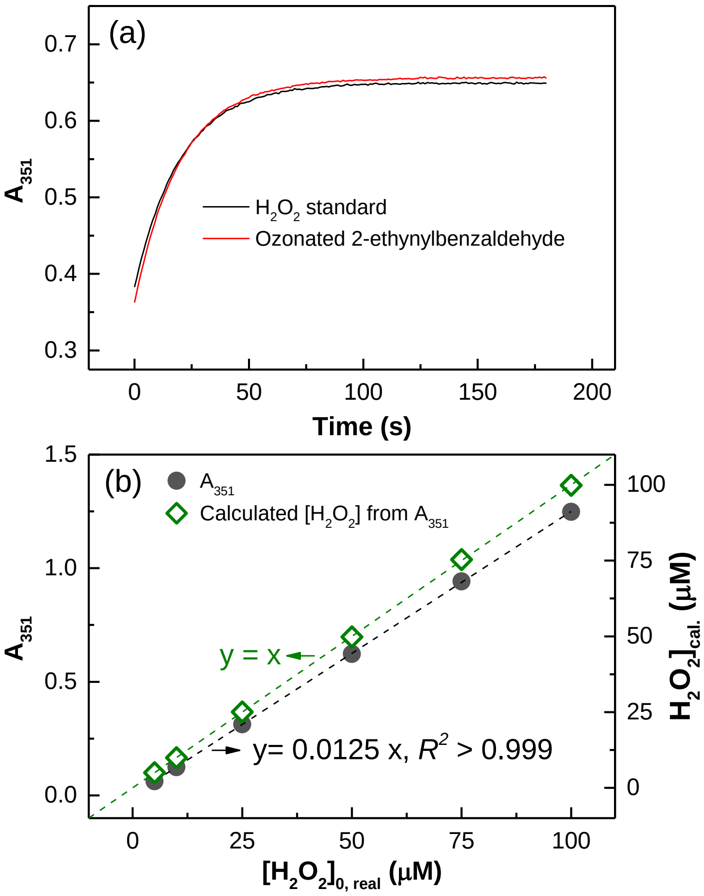

Figure S6. Determination of H_2O_2 concentrations formed during ozonation of 2-ethynylbenzaldehyde by Allen's reagent. (a) UV absorbance change at 351 nm (A_{351}) for determination of a H_2O_2 standard and an ozonated 2-ethynylbenzaldehyde sample by the Allen's reagent method, and (b) a plot of A_{351} as a function of the H_2O_2 concentration in the presence of 2-ethynylbenzaldehyde. Experimental conditions: (a) $[H_2O_2]_0$ = 50 μM, [2-ethynylbenzaldehyde]$_0$ = 500 μM, [DMSO]$_0$ = 10 mM, pH 2.3, 10 mM phosphate buffer for the H_2O_2 standard; [2-ethynylbenzaldehyde]$_0$ = 500 μM, $[O_3]_0$ = 491 μM, [DMSO]$_0$ = 10 mM, reaction time = 1 h, pH 2.3, 10 mM phosphate buffer for the ozonated 2-ethynylbenzaldehyde sample; (b) $[H_2O_2]_0$ = 5–100 μM, [2-ethynylbenzaldehyde]$_0$ = 500 μM, [DMSO]$_0$ = 10 mM, pH 2.3, 10 mM phosphate buffer.

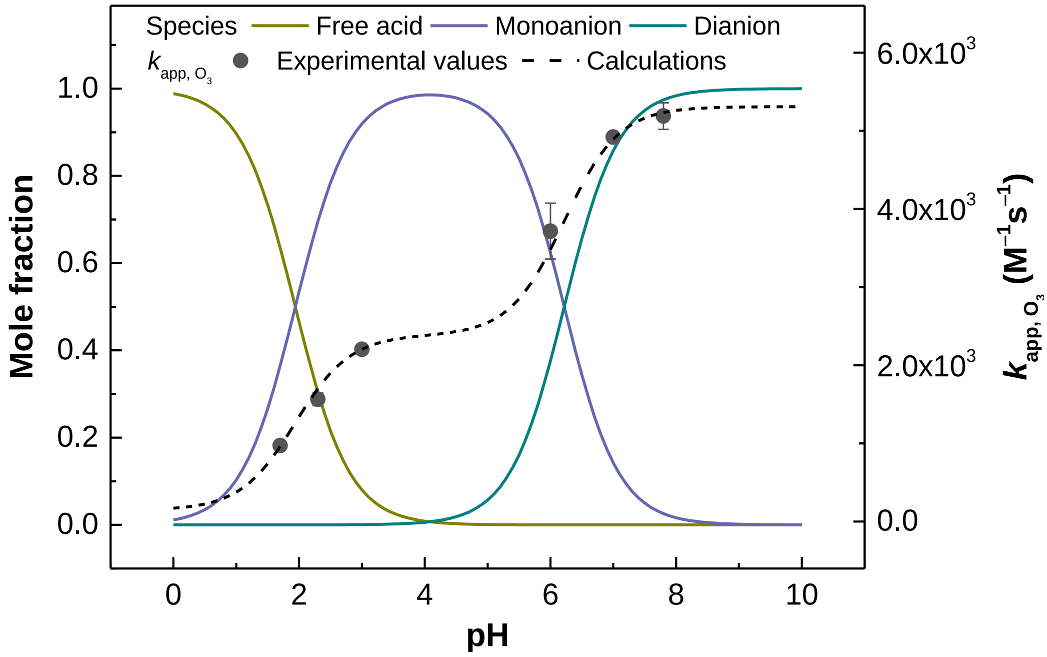

Figure S7. Speciation of maleic acid (mole fraction, solid lines) and the apparent second-order rate constants (k_{app, O_3} , circles (experiments) and dashed line (model calculation)) for its reaction with ozone as a function of the pH. The species-specific second-order rate constants (shown in Table 1) were extrapolated from the k_{app, O_3} values determined at different pH values using a solver program by the generalized reduced gradient non-linear method. They were then used to calculate the pH-dependence of the apparent second-order rate constants (dashed line).

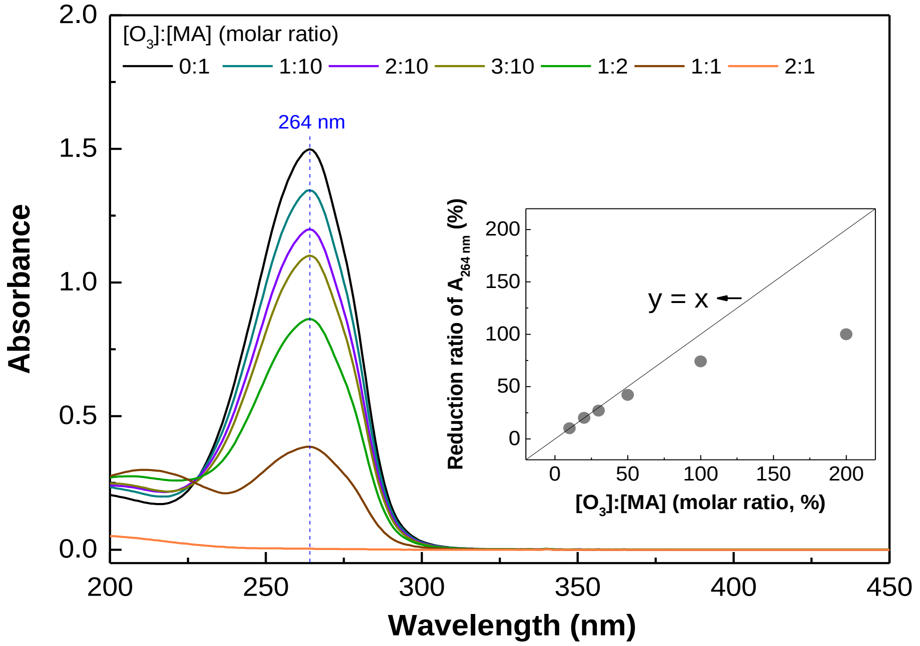

Figure S8. Evolution of UV spectra for ozonated *trans,trans*-muconic acid (MA) samples for different molar ozone:target compound ratios. The insert is a plot of the relative decrease of $A_{264\ nm}$ as a function of the molar ozone:target compound ratio. Experimental conditions: $[MA]_0$ = 50 μM, $[t\text{-BuOH}]_0$ = 5 mM, pH 3.0, 10 mM phosphate buffer.

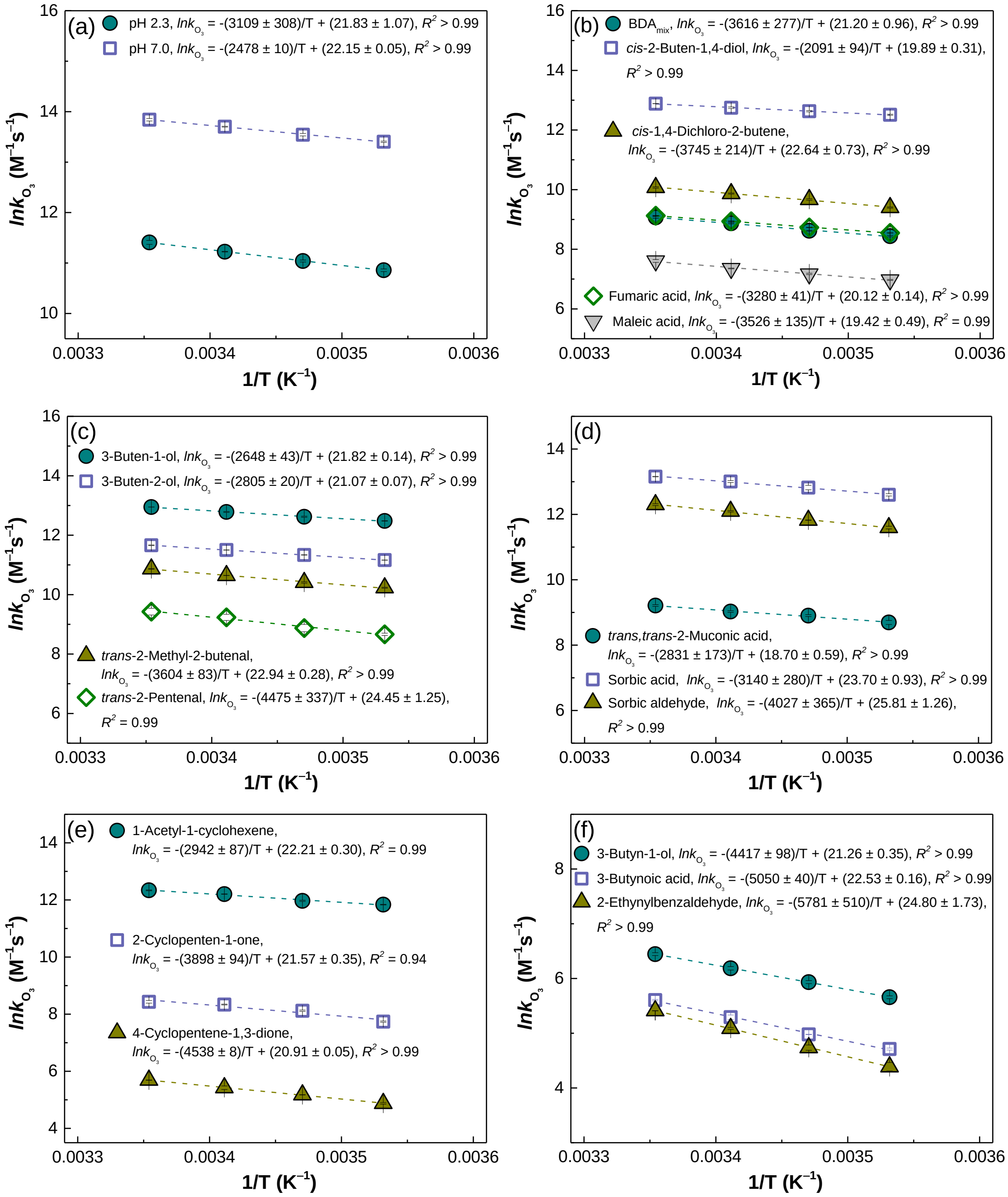

Figure S9. Arrhenius plots for the determination of activation energies for the reactions of ozone with selected olefins and alkynes. (a) Cinnamic acid at pH 2.3 and 7.0, (b) olefins of group I, (c) olefins of group II, (d) olefins of group III, (e) olefins of group IV, and (f) alkynes of group V (Table 1, main text). Dashed lines represent linear regressions. The detailed experimental conditions and determined k_{O_3} values are shown in Table S4, and the resulting activation energies are summarized in Table 1 in the main text. The error bars are the range of the values for different molar ratios of target compound:ozone.

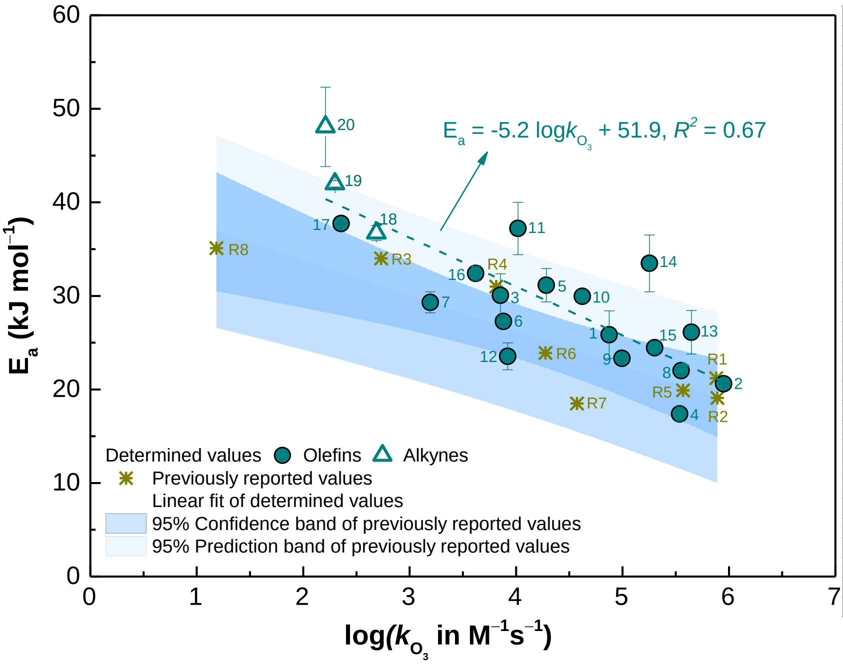

Label	Compound
	Determined compound
1	Cinnamic acid
2	Cinnamic acid (pH 7.0)
3	BDA
4	cis-2-Buten-1,4-diol
5	cis-1,4-Dichloro-2-butene
6	Fumaric acid
7	Maleic acid
8	3-Buten-1-ol
9	3-Buten-2-ol
10	trans-2-Methyl-2-butenal
11	trans-2-Pentenal
12	trans,trans-Muconic acid
13	Sorbic acid
14	Sorbic aldehyde
15	1-Acetyl-1-cyclohexene
16	2-Cyclopenten-1-one
17	4-Cyclopentene-1,3-dione
18	3-Butyn-1-ol
19	3-Butynoic acid
20	2-Ethynylbenzaldehyde
	Previously reported compound
R1	trans-Cinnamic acid (pH 6.5)
R2	trans-Cinnamic acid (pH 7.2)
R3	cis-1,2-Dichloroethene
R4	trans-1,2-Dichloroethene
R5	Isoprene
R6	Methacrolein
R7	Methyl vinyl ketone
R8	Trichloroethene

Figure S10. Previously reported (stars) and currently determined activation energies (E_a) for the reactions of ozone with olefins (circles) and alkynes (triangles) as a function of the logarithm of the corresponding second-order rate constants (k_{O_3}). The currently determined and previously reported values are summarized in Tables 1 and S5, respectively.

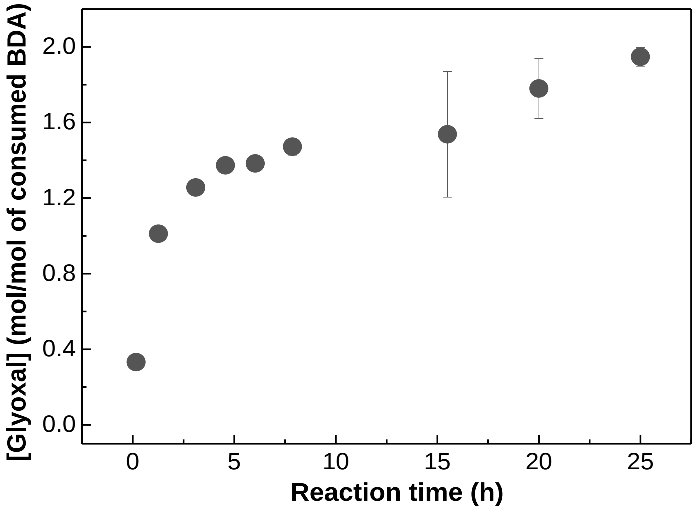

Figure S11. Glyoxal formation from ozonation of BDA as a function of time after complete ozone depletion. Experimental conditions: $[BDA]_0$ = 30 μM, $[O_3]_0$ = 6 and 12 μM for two different experiments, [*t*-BuOH]$_0$ = 3 mM, pH 2.3, 10 mM phosphate buffer. Different from the BDA solution used in Figure 3 in the main text, a 20 mM BDA solution was used here with the following preparation procedure: 14 mL of a 20 mM DMDF aqueous solution was spiked with 1.0 g *p*-toluenesulfonic acid, and then kept at room temperature under magnetic stirring for 6 h. After this contact time, *p*-toluenesulfonic acid was removed by a 0.45 μm syringe filter (CHROMAFIL®Xtra H-PTFE-45/25, Macherey-Nagel, Germany). The circles represent the average values, and the error bars represent the range of values for the two ozone doses.

compound	proposed structure	formula	accurate mass	MS mode
TP 150	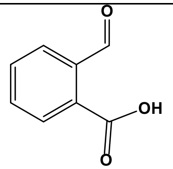	$C_8H_6O_3$	150.0317	negative

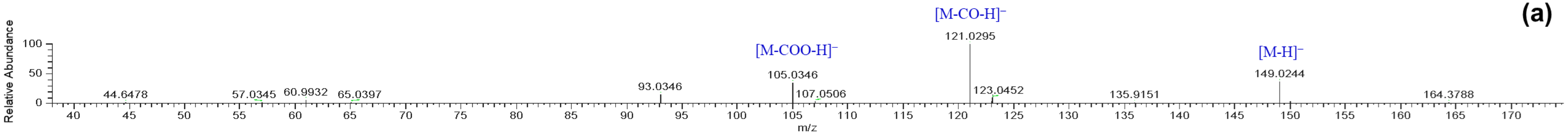

compound	proposed structure	formula	accurate mass	MS mode
TP 134	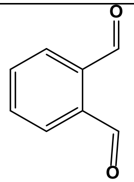	$C_8H_6O_2$	134.0368	positive

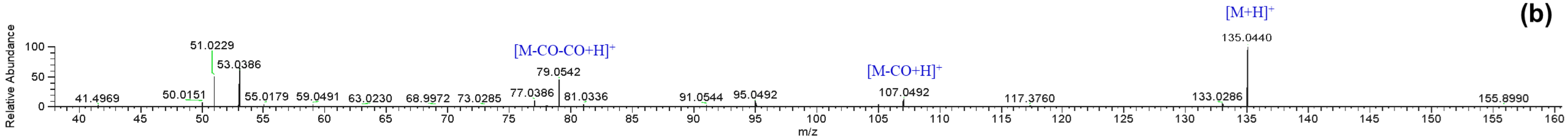

compound	proposed structure	formula	accurate mass	MS mode
TP 162	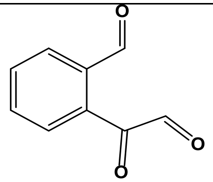	$C_9H_6O_3$	162.0317	positive

(c)

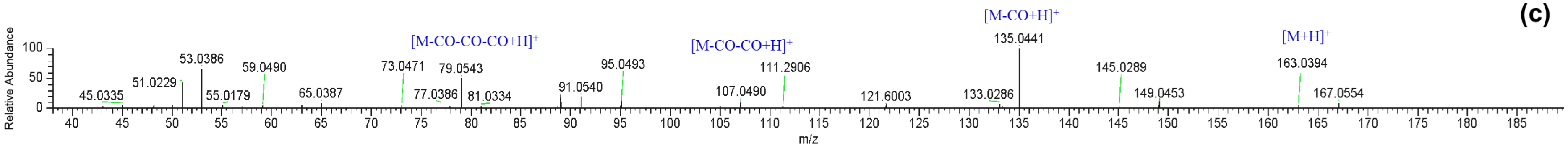

compound	structure	formula	accurate mass	MS mode
2-ethynylbenzalhyde	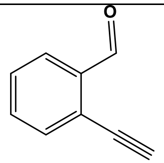	C_9H_6O	130.0419	positive

(d)

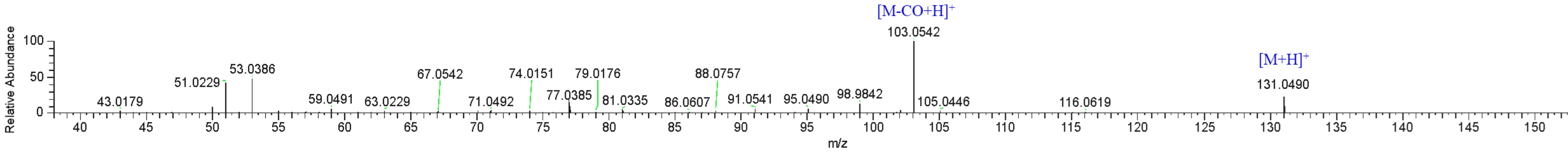

compound	structure	formula	accurate mass	MS mode
2-carboxybenzaldehyde	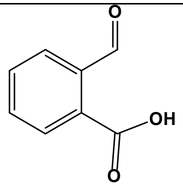	$C_8H_6O_3$	150.0317	negative

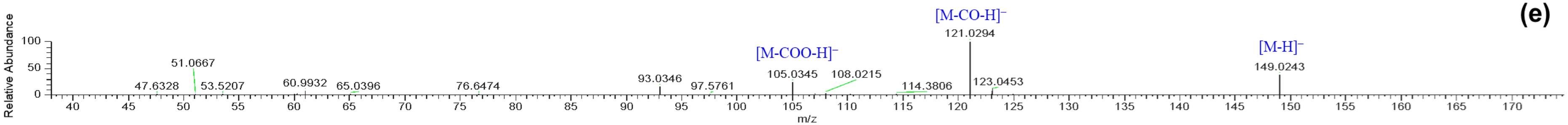

compound	proposed structure	formula	accurate mass	MS mode
phthaldialdehyde	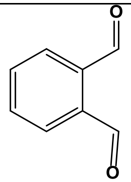	$C_8H_6O_2$	134.0368	positive

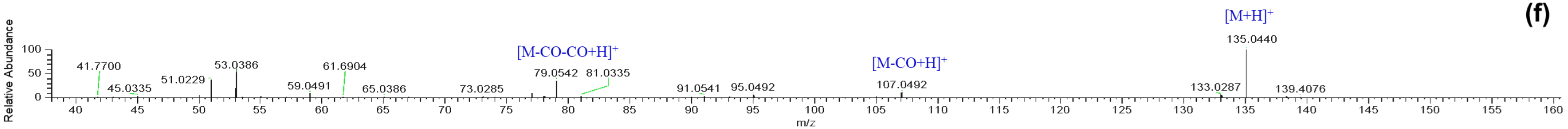

Figure S12. MS^2 spectra of the identified compounds with an Orbitrap LC-MS/MS. (a) TP 150, (b) TP 134, and (c) TP 162 in an ozonated 2-ethynylbenzaldehyde sample. (d) 2-Ethynylbenzaldehyde, (e) 2-carboxybenzaldehyde, and (f) phthaldialdehyde in their standard mixtures.

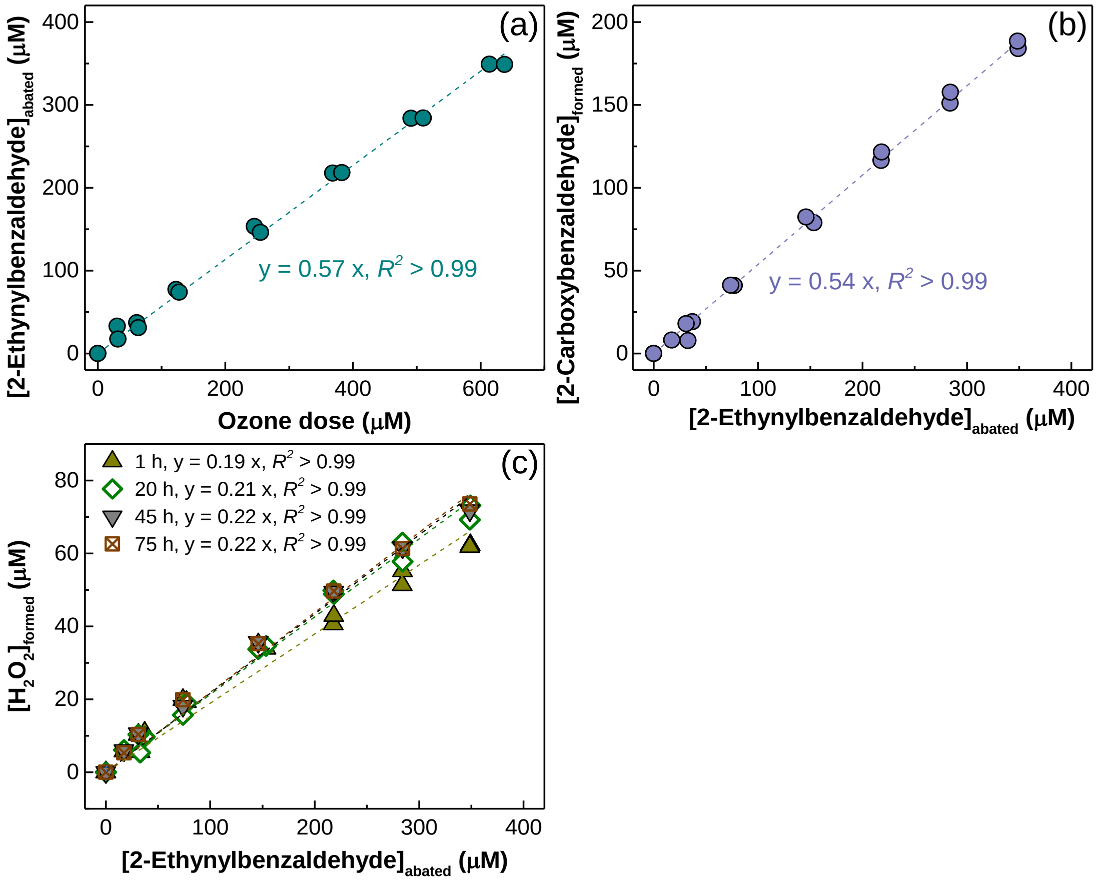

Figure S13. Ozonation of 2-ethynylbenzaldehyde. (a) Abatement of 2-ethynylbenzaldehyde as a function of the ozone dose, (b) formation of 2-carboxybenzaldehyde as a function of the abated 2-ethynylbenzaldehyde, and (c) H_2O_2 formed as a function of the abated 2-ethynylbenzaldehyde. Experimental conditions: $[\text{2-ethynylbenzaldehyde}]_0$ = 500 μM, $[O_3]_0$ = 0–500 μM, $[DMSO]_0$ = 10 mM, pH 2.3, 10 mM phosphate buffer. The samples were diluted by a factor of 2.5 for quantification of 2-carboxybenzaldehyde and 2-ethynylbenzaldehyde.

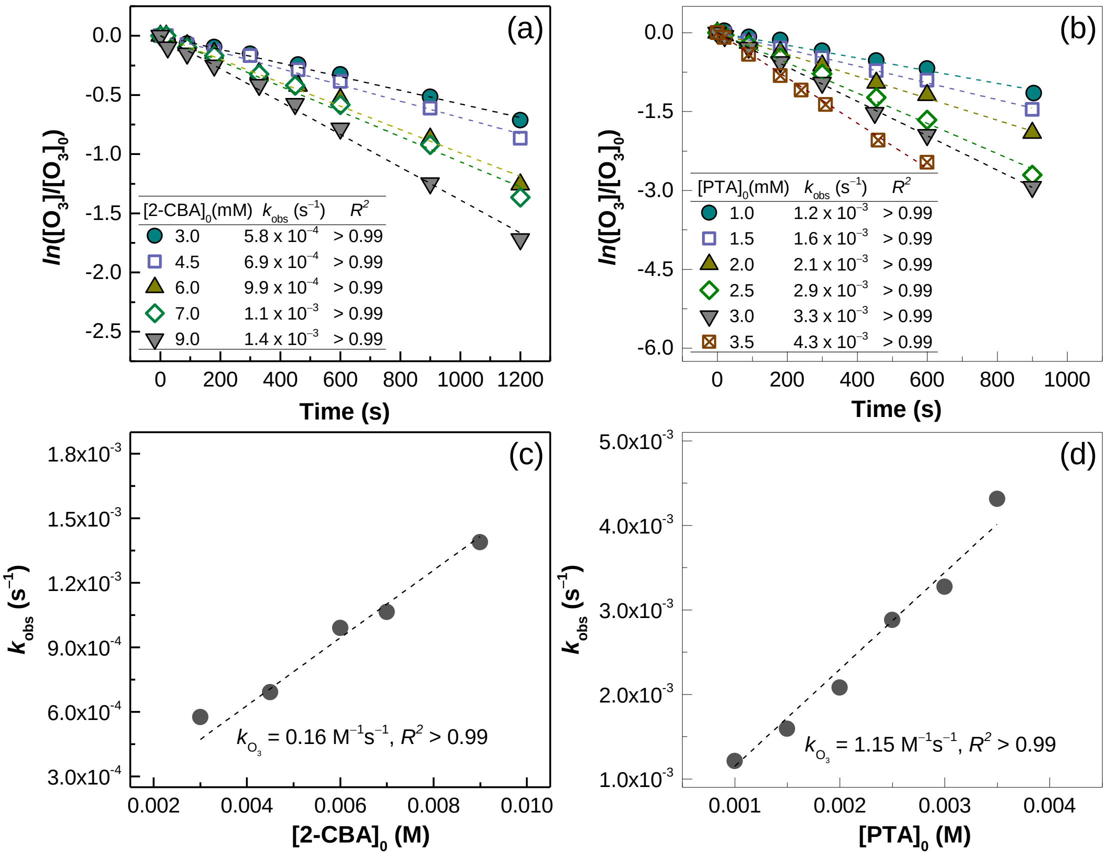

Figure S14. Reactions of ozone with 2-carboxybenzaldehyde (2-CBA) and phthaldialdehyde (PTA). Logarithms of relative ozone residual concentrations in excess of (a) 2-CBA and (b) PTA as functions of time at pH 2.3. Plots of k_{obs} of ozone consumption *versus* the initial concentrations of (c) 2-CBA and (d) PTA. Dashed lines represent linear regressions. The logarithm of the relative ozone residual concentrations was blank-corrected by subtracting the ozone consumption in the absence of 2-CBA and PTA. Experimental conditions: $[TP]_0:[O_3]_0 \geq 10:1$, $[t\text{-BuOH}]_0:[TP]_0 = 10:1$, pH 2.3 ± 0.1, 10 mM phosphate buffer. TP: transformation products, specifically 2-CBA and PTA.

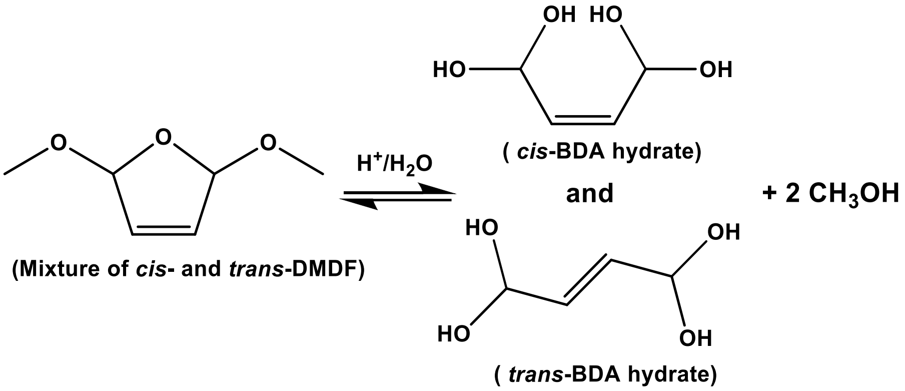

Scheme S1. BDA hydrates formation from DMDF hydrolysis.[52]

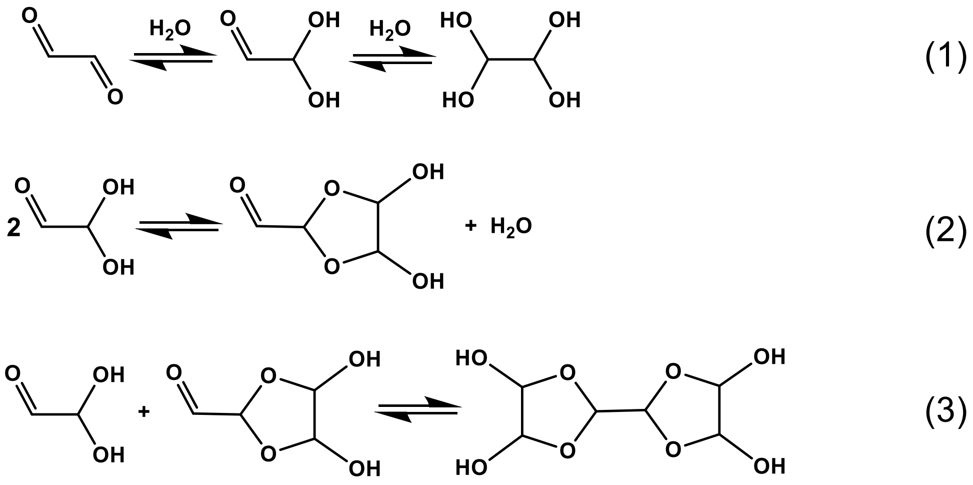

Scheme S2. (1) Hydration and (2, 3) di/trimerization reaction of glyoxal in aqueous solutions.[3,4]

REFERENCES

(1) von Sonntag, C.; von Gunten, U., *Chemistry of ozone in water and wastewater treatment. From basic principles to applications;* IWA: London, 2012.

(2) Whipple, E. B., Structure of glyoxal in water. *J. Am. Chem. Soc.* **1970**, *92* (24), 7183–7186.

(3) Avzianova, E.; Brooks, S. D., Raman spectroscopy of glyoxal oligomers in aqueous solutions. *Spectrochim. Acta, Part A* **2013**, *101*, 40–48.

(4) Loeffler, K. W.; Koehler, C. A.; Paul, N. M.; De Haan, D. O., Oligomer formation in evaporating aqueous glyoxal and methyl glyoxal solutions. *Environ. Sci. Technol.* **2006**, *40* (20), 6318–6323.

(5) Stocco, L., Quantification of ozone-reactive precursors in dissolved organic matter: Formation of H_2O_2 and its transformation to O_2 for isotope ratio analysis, Master's Thesis EPFL, 2021. https://infoscience.epfl.ch/record/295199.

(6) Klassen, N. V.; Marchington, D.; McGowan, H. C. E., H_2O_2 Determination by the I_3^- Method and by $KMnO_4$ Titration. *Anal. Chem.* **1994**, *66* (18), 2921–2925.

(7) Bader, H.; Sturzenegger, V.; Hoigné, J., Photometric method for the determination of low concentrations of hydrogen peroxide by the peroxidase catalyzed oxidation of N,N-diethyl-*p*-phenylenediamine (DPD). *Water Res.* **1988**, *22* (9), 1109–1115.

(8) Hoigné, J.; Bader, H., Characterization of water quality criteria for ozonation processes. Part II: Lifetime of added ozone. *Ozone: Sci. Eng.* **1994**, *16* (2), 121–134.

(9) Bader, H.; Hoigné, J., Determination of ozone in water by the indigo method. *Water Res.* **1981**, *15* (4), 449–456.

(10) Gottlieb, H. E.; Kotlyar, V.; Nudelman, A., NMR chemical shifts of common laboratory solvents as trace impurities. *J. Org. Chem.* **1997**, *62* (21), 7512–7515.

(11) Elovitz, M. S.; von Gunten, U.; Kaiser, H. P., Hydroxyl radical/ozone ratios during ozonation processes. II. The effect of temperature, pH, alkalinity, and DOM properties. *Ozone: Sci. Eng.* **2000**, *22* (2), 123–150.

(12) Driedger, A.; Staub, E.; Pinkernell, U.; Marinas, B.; Koster, W.; von Gunten, U., Inactivation of *Bacillus subtilis* spores and formation of bromate during ozonation. *Water Res.* **2001**, *35* (12), 2950–2960.

(13) Pitzer, K. S.; Hollenberg, J. L., *cis*- and *trans*-Dichloroethylenes. The infrared spectra from 130–400 $Cm.^{-1}$ and the thermodynamic properties[1]. *J. Am. Chem. Soc.* **1954**, *766* (6), 4.

(14) Chaudhuri, R. K.; Hammond, J. R.; Freed, K. F.; Chattopadhyay, S.; Mahapatra, U. S., Reappraisal of *cis* effect in 1,2-dihaloethenes: An improved virtual orbital multireference approach. *J. Chem. Phys.* **2008**, *129* (6), 064101.

(15) *Handbook of Chemistry and Physics*, 71st. edn.; Lide, D. K., Ed.; CRC Press: Boca Raton, FL, 1991; pp: 8–35.

(16) National center for biotechnology information. Pubchem compound summary for CID 444972, fumaric Acid. https://pubchem.ncbi.nlm.nih.gov/compound/Fumaric-Acid (accessed Nov 10, 2023).

(17) National center for biotechnology information. Pubchem compound summary for CID 444266, maleic acid. https://pubchem.ncbi.nlm.nih.gov/compound/Maleic-Acid (accessed Nov 10, 2023).

(18) Ramseier, M. K.; von Gunten, U., Mechanisms of phenol ozonation—kinetics of formation of primary and secondary reaction products. *Ozone: Sci. Eng.* **2009**, *31* (3), 201–215.

(19) Lück, E.; Jager, M.; Raczek, N., *In Ullmann's encyclopedia of industrial chemistry*, 6th ed.; Wiley-VCH Verlag GmbH & Co. KGaA: Weinheim, Germany, 2005.

(20) Chemical book. CAS database list for CAS No. 2345-51-9, 3-butynoic acid. https://www.chemicalbook.com/ChemicalProductProperty_EN_CB4500119.htm (accessed Nov 10, 2023).

(21) Buxton, G. V.; Greenstock, C. L.; Helman, W. P.; Ross, A. B., Critical Review of rate constants for reactions of hydrated electrons, hydrogen atoms and hydroxyl radicals (•OH/•O^-) in Aqueous Solution. *J. Phys. Chem. Ref. Data* **1988**, *17* (2), 374.

(22) Yao, C. C. D.; Haag, W. R., Rate constants for direct reactions of ozone with several drinking-water contaminants. *Water Res.* **1991**, *25* (7), 761–773.

(23) Leitzke, A.; von Sonntag, C., Ozonolysis of unsaturated acids in aqueous solution: Acrylic, methacrylic, maleic, fumaric and muconic acids. *Ozone: Sci. Eng.* **2009**, *31* (4), 301–308.

(24) Leitzke, A.; Flyunt, R.; Theruvathu, J. A.; von Sonntag, C., Ozonolysis of vinyl compounds, CH_2=CH-X, in aqueous solution—the chemistries of the ensuing formyl compounds and hydroperoxides. *Org. Biomol. Chem.* **2003**, *1* (6), 1012–1019.

(25) Hoigné, J.; Bader, H., Rate constants of reactions of ozone with organic and inorganic compounds in water—I: non-dissociating organic compounds. *Water Res.* **1983**, *17* (2), 173–183.

(26) Mvula, E.; von Sonntag, C., Ozonolysis of phenols in aqueous solution. *Org.*

Biomol. Chem. **2003**, *1* (10), 1749–1756.

(27) Dowideit, P.; von Sonntag, C., Reaction of ozone with ethene and its methyl- and chlorine-substituted derivatives in aqueous solution. *Environ. Sci. Technol.* **1998**, *32* (8), 1112–1119.

(28) Theruvathu, J. A.; Flyunt, R.; Aravindakumar, C. T.; von Sonntag, C., Rate constants of ozone reactions with DNA, its constituents and related compounds. *J.. Chem. Soc., Perkin Trans. 2* **2001**, (3), 269–274.

(29) Witkowski, B.; Al-Sharafi, M.; Gierczak, T., Ozonolysis of *β*-caryophyllonic and limononic acids in the aqueous phase: Kinetics, product yield, and mechanism. *Environ. Sci. Technol.* **2019**, *53* (15), 8823–8832.

(30) Wolf, C.; von Gunten, U.; Kohn, T., Kinetics of inactivation of waterborne enteric viruses by ozone. *Environ. Sci. Technol.* **2018**, *52* (4), 2170–2177.

(31) Witkowski, B.; Al-Sharafi, M.; Gierczak, T., Kinetics of limonene secondary organic aerosol oxidation in the aqueous phase. *Environ. Sci. Technol.* **2018**, *52* (20), 11583–11590.

(32) Kim, M. S.; Lee, C., Ozonation of microcystins: Kinetics and toxicity decrease. *Environ. Sci. Technol.* **2019**, *53* (11), 6427-6435.

(33) Leitzke, A.; Reisz, E.; Flyunt, R.; von Sonntag, C., The reactions of ozone with cinnamic acids: Formation and decay of 2-hydroperoxy-2-hydroxyacetic acid. *J. Chem. Soc., Perkin Trans. 2* **2001**, (5), 793–797.

(34) Jans, U., Radikalbildung aus Ozon in atmosphärischen Wassern - Einfluss von Licht, gelösten Stoffen und Russpartikeln. Doctoral Thesis ETHZ 11814, 1996.

(35) Peter, A.; von Gunten, U., Oxidation kinetics of selected taste and odor compounds during ozonation of drinking water. *Environ. Sci. Technol.* **2007**, *41* (2), 626–631.

(36) Keady, H. D.; Kuo, C. H., A data acquisition system for rapid kinetic experiments. *Chem. Eng.. Commun.* **1983**, *23* (4–6), 291–304.

(37) Hoigné, J.; Bader, H., Rate constants of reactions of ozone with organic and inorganic compounds in water—II: dissociating organic compounds. *Water Res.* **1983**, *17* (2), 185–194.

(38) Benbelkacem, H.; Mathé, S.; Benbelkacem, H., Taking mass transfer limitation into account during ozonation of pollutants reacting fairly quickly. *Water Sci. Technol.* **2004**, *49* (4), 25–30.

(39) Lee, M.; Merle, T.; Rentsch, D.; Canonica, S.; von Gunten, U., Abatement of

polychoro-1,3-butadienes in aqueous solution by ozone, UV photolysis, and advanced oxidation processes (O_3/H_2O_2 and UV/H_2O_2). *Environ. Sci. Technol.* **2017**, *51* (1), 497–505.

(40) Pryor, W. A.; Giamalva, D. H.; Church, D. F., Kinetics of ozonation. 2. Amino acids and model compounds in water and comparisons to rates in nonpolar solvents. *J. Am. Chem. Soc.* **1984**, *106* (23), 7094–7100.

(41) Pedersen, T.; Sehested, K., Rate constants and activation energies for ozonolysis of isoprene methacrolein and methyl-vinyl-ketone in aqueous solution: Significance to the in-cloud ozonation of isoprene. *Int. J. Chem. Kinet.* **2001**, *33* (3), 182–190.

(42) Witkowski, B.; Jurdana, S.; Gierczak, T., Limononic acid oxidation by hydroxyl radicals and ozone in the aqueous phase. *Environ. Sci. Technol.* **2018**, *52* (6), 3402–3411.

(43) Benbelkacem, H.; Cano, H.; Mathe, S.; Debellefontaine, H., Maleic acid ozonation: Reactor modeling and rate constants determination. *Ozone: Sci. Eng.* **2003**, *25* (1), 13–24.

(44) Beltrán, F. J.; Rodríguez, E. M.; Romero, M. T., Kinetics of the ozonation of muconic acid in water. *J. Hazard. Mater.* **2006**, *138* (3), 534–538.

(45) Onstad, G. D.; Strauch, S.; Meriluoto, J.; Codd, G. A.; von Gunten, U., Selective oxidation of key functional groups in cyanotoxins during drinking water ozonation. *Environ. Sci. Technol.* **2007**, *41* (12), 4397–4404.

(46) Leviss, D. H.; Van Ry, D. A.; Hinrichs, R. Z., Multiphase ozonolysis of aqueous *α*-Terpineol. *Environ. Sci. Technol.* **2016**, *50* (21), 11698–11705.

(47) Beltrán, F. J.; González, M.; Rivas, J.; Tierno, M., Elimination pathways during water ozonation of volatile organochlorine compounds. *Toxicol. Environ. Chem.* **1997**, *63* (1–4), 107–118.

(48) Huber, M. M.; Ternes, T. A.; von Gunten, U., Removal of estrogenic activity and formation of oxidation products during ozonation of 17α-ethinylestradiol. *Environ. Sci. Technol.* **2004**, *38* (19), 5177–5186.

(49) Tekle-Röttering, A.; Lim, S.; Reisz, E.; Lutze, H. V.; Abdighahroudi, M. S.; Willach, S.; Schmidt, W.; Tentscher, P. R.; Rentsch, D.; McArdell, C. S.; Schmidt, T. C.; von Gunten, U., Reactions of pyrrole, imidazole, and pyrazole with ozone: kinetics and mechanisms. *Environ. Sci. Water Res. Technol.* **2020**, *6* (4), 976–992.

(50) Zoumpouli, G. A.; Zhang, Z. Y.; Wenk, J.; Prasse, C., Aqueous ozonation of furans: Kinetics and transformation mechanisms leading to the formation of α,β-

unsaturated dicarbonyl compounds. *Water Res.* **2021**, *203*, 117487.

(51) Huber, M. M.; Canonica, S.; Park, G. Y.; von Gunten, U., Oxidation of pharmaceuticals during ozonation and advanced oxidation processes. *Environ. Sci. Technol.* **2003**, *37* (5), 1016–1024.

(52) Hansen, E. W.; Holm, K. H.; Jahr, D. M.; Olafsen, K.; Stori, A., Reaction of poly(vinyl alcohol) and dialdehydes during gel formation probed by ^{1}H n.m.r.—a kinetic study. *Polymer* **1997**, *38* (19), 4863–4871.
